# Variable outcomes of human heart attack recapitulated in genetically diverse mice

**DOI:** 10.1038/s41536-019-0067-6

**Published:** 2019-03-04

**Authors:** Ekaterina Salimova, Kristen J. Nowak, Ana C. Estrada, Milena B. Furtado, Elyshia McNamara, Quang Nguyen, Lois Balmer, Christoph Preuss, Jeffrey W. Holmes, Mirana Ramialison, Grant Morahan, Nadia A. Rosenthal

**Affiliations:** 1 Australian Regenerative Medicine Institute, Monash University, Clayton, VIC Australia; 2 Monash Biomedical Imaging, Monash University, Clayton, VIC Australia; 3 Faculty of Health and Medical Sciences, School of Biomedical Sciences, The University of Western Australia, Perth, WA Australia; 4 QEII Medical Centre, Nedlands and Centre for Medical Research, Harry Perkins Institute of Medical Research, The University of Western Australia, Perth, WA Australia; 5 Office of Population Health Genomics, Division of Public and Aboriginal Health, Western Australian Department of Health, East Perth, WA Australia; 6 Departments of Biomedical Engineering and Medicine, and Robert M. Berne Cardiovascular Research Center, University of Virginia, Charlottesville, VA USA; 7 The Jackson Laboratory, Bar Harbor, ME USA; 8 School of Medical and Health Science, Edith Cowan University, Joondalup, Australia; 9 National Heart and Lung Institute, Imperial College London, London, UK

## Abstract

Clinical variation in patient responses to myocardial infarction (MI) has been difficult to model in laboratory animals. To assess the genetic basis of variation in outcomes after heart attack, we characterized responses to acute MI in the Collaborative Cross (CC), a multi-parental panel of genetically diverse mouse strains. Striking differences in post-MI functional, morphological, and myocardial scar features were detected across 32 CC founder and recombinant inbred strains. Transcriptomic analyses revealed a plausible link between increased intrinsic cardiac oxidative phosphorylation levels and MI-induced heart failure. The emergence of significant quantitative trait loci for several post-MI traits indicates that utilizing CC strains is a valid approach for gene network discovery in cardiovascular disease, enabling more accurate clinical risk assessment and prediction.

## Cardiology: Genetically diverse mice show human-like responses to heart attack

Mice from a genetically diverse panel of inbred strains show a variety of biological outcomes after a heart attack (myocardial infarction), just as humans do. This ‘Collaborative Cross’ mouse resource—which is already widely used in other disciplines of biomedical research—thus provides a tractable system for investigating the genetic factors contributing to acute and chronic presentations of heart disease. Ekaterina Salimova from Monash University in Clayton, Australia, and colleagues experimentally induced myocardial infarctions in the 32 founder or recombinant strains from the Collaborative Cross. They documented large differences in survival, cardiac dilation and scar size among different strains. Gene expression profiling and quantitative trait locus mapping revealed a large number of candidate genes and molecular pathways linked to adverse outcomes. These could offer promising drug targets for treating the damage wrought by heart attacks.

## Introduction

The mortality rate among patients having heart failure (HF) with reduced ejection fraction (EF) following a heart attack has gradually been reduced through the cumulative benefit of medications such as angiotensin-converting enzyme inhibitors, angiotensin-receptor blockers, β-blockers, and mineralocorticoid-receptor antagonists.^1,2^ However, surviving patients with ischemic cardiomyopathy and HF face a broad spectrum of symptoms, emphasizing the need for new prognostic models to stratify patient subgroups and to develop new therapies. Predominant risk predictors include genetic factors, which have a profound influence on incidence and outcome of cardiovascular disease.^3–5^

Mice have provided an effective experimental system to study the general pathophysiology of heart disease, to identify molecular markers and to assess therapeutic options. Inbred mouse models comprise useful experimental systems for functional analysis of genetic variants in the context of disease phenotyping, with validation in controlled conditions, high reproducibility, and unlimited access to tissues for the identification of genes contributing to simple Mendelian traits. Even so, experimental strategies for complex trait analyses in the mouse have not kept pace with rapid developments in human genetic studies, and thus new strategies for using model organisms are needed. A major drawback of mouse models has been the limited genetic diversity in inbred laboratory mouse strains, which do not accurately capture the complex genetics of the human population. With over 20,000 interacting mammalian genes, each with multiple variants, genetically diverse experimental mouse systems have proven a more powerful tool to dissect cardiovascular features.^6^ Such systems are essential for building predictive models of predisposing traits for outcomes with such a complex etiology as cardiovascular disease and for making precision medicine a reality.

The Collaborative Cross (CC) multi-parental recombinant inbred (RI) panel was specifically designed for performing gene association studies of complex human disease, with increased requisite statistical power and resolution for mapping complex traits that better reflects clinical genetic variation.^7,8^ The CC panel encompasses multiple RI mouse strains derived from eight founder inbred mouse lines (A/J, C57BL/6J, 129S1/SvImJ, NOD/ShiLtJ, NZO/H1LtJ, CAST/EiJ, PWK/PhJ, and WSB/EiJ), in which all strains inherit different founder genome components through randomization. The CC panel offers a tractable experimental system designed for the correlation of phenotypes with underlying biomolecular, physiological, and morphological characteristics. The CC harnesses over 90% of common genetic variation of the mouse species. In addition, genetic variation in CC RI strains is uniformly distributed, with multiple allelic variants in the coding or regulatory regions of essentially all known genes, in contrast to classical inbred strains of mice, which have limited genetic diversity.^9^ The genetic architecture of the CC population has been derived from complete genome sequencing of 8 CC founder and 69 RI strains, revealing high allelic diversity and affording precision mapping of phenotypic quantitative trait loci (QTL).^10^ The CC panel serves as a resource of stable genotypes for mechanistic analysis: each CC inbred strain represents a fixed and reproducible genotype, providing retrievable, genome-matched mice for experimental replication, trait correlation, and mapping by association analysis, affording resolution at least an order of magnitude better than that achieved by traditional linkage methods. The CC also provides models of diseases not previously available in the mouse, e.g., diabetic retinopathy.^11^

Reasoning that the CC panel could provide valuable insight into the genetic complexity of cardiovascular disease, we carry out a cardiac susceptibility pilot screen by subjecting cohorts of CC strains to experimental myocardial infarction (MI)^12^ and evaluating multiple parameters of injury response. Dramatic variation in MI survival, cardiac dilation and scar size is scored in 32 CC founder and RI lines. Cardiac gene expression profiling reveals enrichment in metabolic features for specific traits, whilst genetic analysis demonstrates distinct loci for parameters such as cardiac rupture and HF (left ventricular dilation). Collectively, these results highlight the variation in genetic factors influencing both acute and chronic presentations of heart disease, and provide new, genetically tractable models for dissecting the diversity of human cardiomyopathies.

## Results

### Variation in heart morphology and function across CC mouse strains

A preliminary survey of CC founder and RI strains revealed marked differences in various morphological parameters, e.g., coat color and quality, body weight (BWt), behavior, neurological activity, and bleeding (unpublished results), in accordance with previous reports on variation in aspects such as motor performance,^13^ hematological parameters,^14^ susceptibility to infections,^15–17^ immunological conditions,^18,19^ reproduction,^20^ toxicokinetics,^21^ glycome repertoire,^22^ and traits associated with skin cancer.^23^

**Fig. 1:**
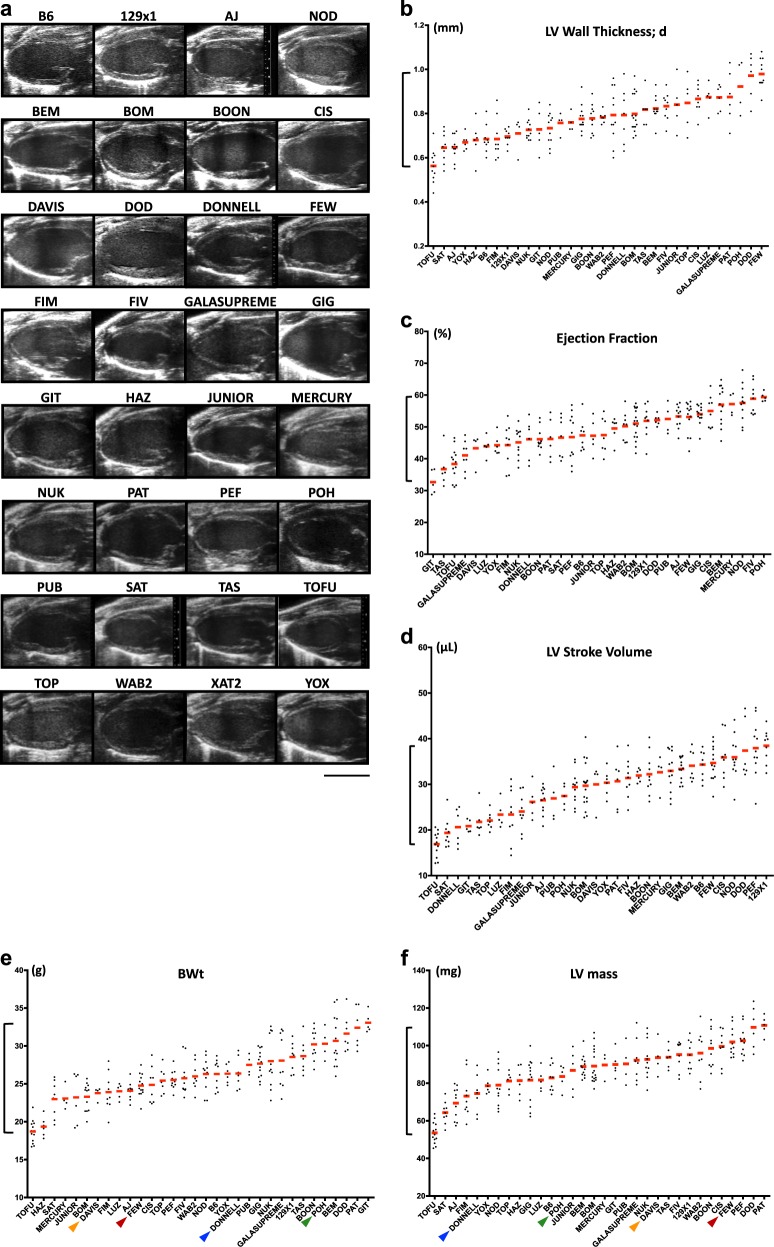
Comparative morphological and functional analysis of selected CC RI strains (12-week-old male mice). **a** Representative echographic images of the left ventricles in longitudinal axis. **b**–**f** Plots showing distribution of individual values among RI strains for **b** LV wall thickness in diastole, **c** ejection fraction, **d** LV stroke volume, **e** body weight (BWt), and **f** LV mass (calculations based on echographic measurements). Colored arrows point at the strains with most prominent differences between BWt and LV weight. Scale bar, 5 mm **a**

To assess parameters of cardiac morphology and function, echocardiographic analysis was performed on 12-week-old male mice from various CC RI strains, with particular focus on LV function. We documented significant variation in LV morphology (Fig. 1a) as well as other parameters, exemplified by LV wall thickness, EF, and stroke volume (SV) (Fig. 1b–d). It was notable that in certain CC RI strains LV mass did not show direct correlation with BWt (Fig. 1e, f), as exemplified by the DAVIS_BA, DONNELL_HA, FEW_FD, and POH_DC strains (colored arrows). Although most strains had steady heart rates of 400–450 bpm during ultrasound imaging under standard conditions (1.5% v/v isoflurane anesthesia and a body temperature of 37 °C), some strains revealed a tendency to maintain a stably higher (>500 bpm, e.g., BEM_AG, DONNELL_HA, FEW_FD, POH_DC) or a stably lower (<400 bpm, e.g., 129 × 1, NOD/ShiLtJ, PEF_EC) heart rate (data not shown). These findings underscore the reproducible variability among CC RI strains in virtually every parameter measured. A complete summary of measurements and calculations is outlined in Table S1 (Supplementary Material).

### Strain variation in perioperative survival after MI

**Fig. 2:**
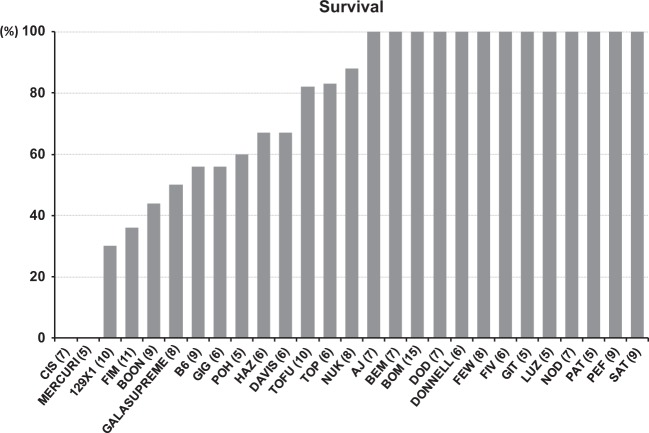
Susceptibility of selected CC strains to myocardial rupture. Survival rate of mice 3–5 days after MI corresponding to mortality due to myocardial rupture. Only animals with > 30% LV ischemia were included in the calculation. Initial number of animals per strain is indicated in brackets next to the strain name (*n*)

After baseline ultrasound examination, mice were subjected to left coronary artery (LCA) ligation surgery to model MI. Although all assessed strains recovered normally immediately after surgery, there was a significant difference in survival 3–5 days post MI (Fig. 2). This survival drop-off prominent in some strains was due to death by myocardial rupture, a typical feature of the mouse MI model. Notably, the C57BL/6J (B6) parental strain (most widely used in cardiovascular research) displayed one of the highest mortality rates. Two CC RI strains (CIS_AD and MERCURI_HF) had 100% penetrance for this trait.

During surgical procedures, there were distinct morphological differences among CC RI strains in trachea diameter and/or LCA position and branching. Narrow/fragile trachea impeded successful intubation, whereas early LCA branching and/or atypical position was a major hindrance for obtaining a reproducible infarct size. Thereafter, strains exhibiting such phenotypes were excluded from further analysis (e.g., PUB_CD, TAS_FE, and YOX_DE). For other strains, only those with a comparably large MI size (~40% of LV) were included for further study; as from previous studies^24^ and our observations, an MI size of <30% does not result in significant reduction of EF and LV remodeling.

### Post-MI diversity in heart function and morphology

**Fig. 3:**
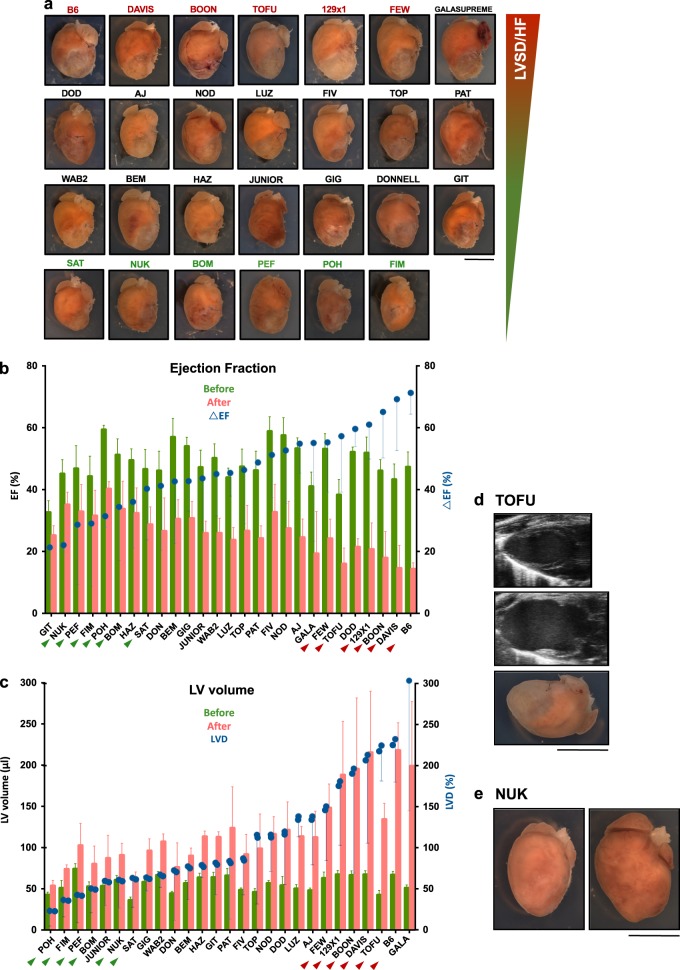
Morphological and functional analysis of the hearts one month after MI. **a** Representative images of the hearts of selected CC RI strains one month after MI, aligned from the most pronounced (top, in red) to the least pronounced (bottom, in green) LVSD/HF phenotype. **b** Changes in EF for individual strains (green bars—before, red bars—1 m after MI). Strains are aligned according to the degree of EF reduction after MI (ΔEF—blue dots). **c** LV volume changes (green bars—before, red bars—1 m after MI). Strains are aligned according to the degree of LV dilation after MI (LVD—blue dots). Green and red arrows on *X* axis indicate the strains with correlating ΔEF and LVD values (LVSD/HF phenotype). Error bars on the graphs denote standard deviation. Data are presented as mean value ± SD of minimum three biological replicates. (Exact number biological replicates per strain is provided in Supplementary Tables S1 and S2.) **d** TOFU_FB strain as a potential model of MI-induced dilated cardiomyopathy. Representative echographic images of the LV in longitudinal axis before (top) and after MI (middle), whole-mount image of the same heart one month after MI (bottom). **e** NUK_AC strain as a potential model of spontaneous right ventricular hypertrophy. Normal heart (left), heart with severe RV hypertrophy (right). Scale bars, 5 mm (**a**, **d**)

Heart function of the surviving mice was assessed by echocardiography 1 month after MI surgery, at which point hearts were collected and whole-mount images obtained (Fig. 3a). As with baseline measurements, there was dramatic variation in the outcome of MI between strains. Considering that reduced EF (ΔEF) and LV dilation (LVD) are the key hallmarks of MI-induced LV systolic dysfunction (LVSD) and subsequent HF,^1,25,26^ these two parameters were assessed for each strain before and after MI. Significant variation was detected between the strains, with B6 being one of the worst performing strains (Fig. 3b, c).

Strains displaying severe and minimal LVSD based on the combination of ΔEF and LVD traits are highlighted on Fig. 3a in red and green, respectively. Notably, some strains (e.g., GIT_GC, GALASUPREME_CE, JUNIOR_GB, DOD_AH, HAZ_EF) did not show evidence of a direct correlation between ΔEF and LVD traits. For the consistency of the study, these were excluded from LVSD/HF-related analysis. A complete summary of post-MI measurements and calculations is presented in Table S2 (Supplementary Material).

Of all the strains assessed, TOFU_FB exemplified an excellent model of MI-induced dilated cardiomyopathy with 100% survival, high phenotypic penetrance, and reproducibility of post-MI response. Multiple animals of the NUK_AC strain manifested various cardiac abnormalities in homeostatic condition, including heart and right ventricular hypertrophy. These individual animals were not included in this study (Fig. 3d).

**Fig. 4:**
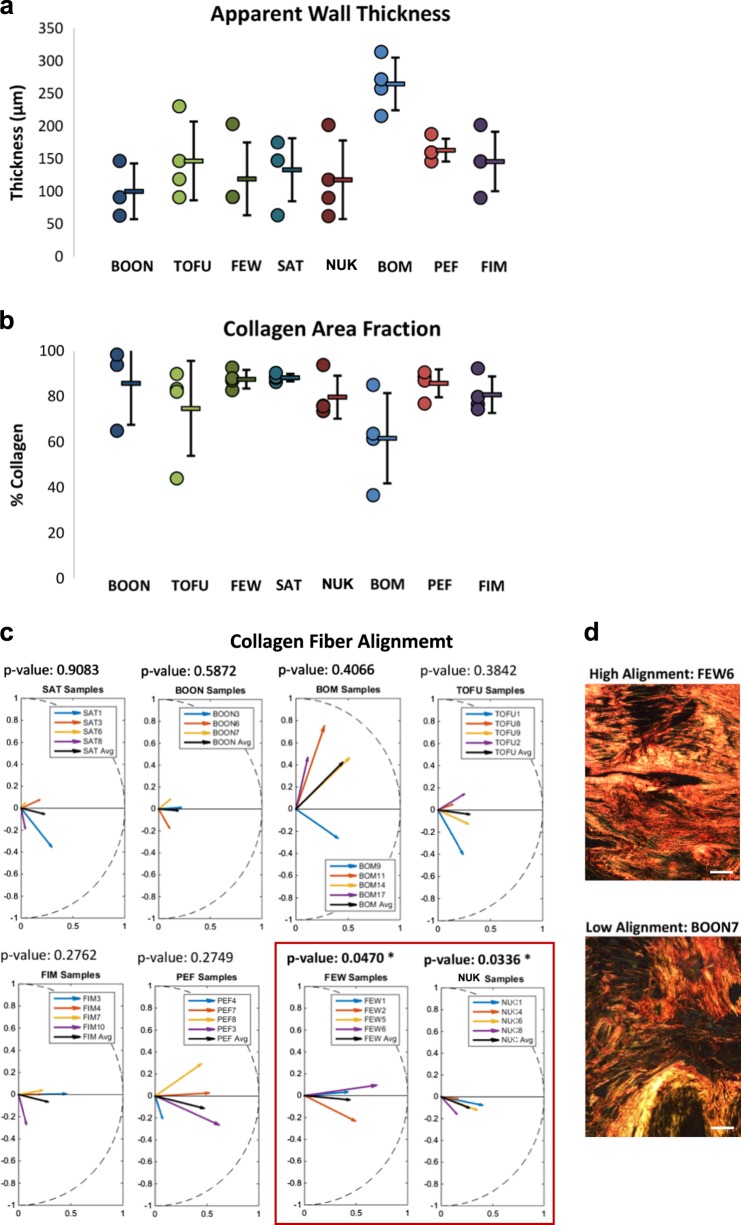
Comparative morphological and qualitative analysis of the LV scars one month after MI. **a** Scar wall thickness. **b** Collagen area fraction. **c** Analysis of collagen fiber alignment. The FEW_FD and NUK_AC strains show consistent alignment as determined by a one-sample *t*-test. **d** Representative images of well-aligned (top) and poorly aligned (bottom) collagen fibers (polarized microscopy). Scale bar, 100 µm **d**

Reasoning that scar quality and composition might correlate with LVD, scar analysis was performed on eight selected CC RI strains, with differentially pronounced LVSD/HF phenotype. Significant differences in apparent wall thickness in the embedded scar samples ranged from an average of 100 µm for the BOON_HF strain to over 250 µm for the BOM_GB strain (Fig. 4a). The collagen area fraction (AF) was high in most strains, with substantial inter-sample variability (Fig. 4b). Two of the CC RI strains (FEW_FD and NUK_AC) showed significant collagen fiber alignment (Fig. 4c, d) and in both cases the average orientation of the collagen fibers was close to the circumferential direction. Most other strains showed collagen alignment patterns more similar to that reported following coronary artery ligation in rats.^27^ Individual scars often displayed substantial alignment as indicated by a mean vector length (MVL) of 0.4 or higher, but the group did not display significant alignment on average, because the mean orientation varied between samples. In summary, whereas collagen content was similar across strains, strength of collagen alignment varied widely.

### Distinct genetics of heart repair vs ear hole punch healing

**Fig. 5:**
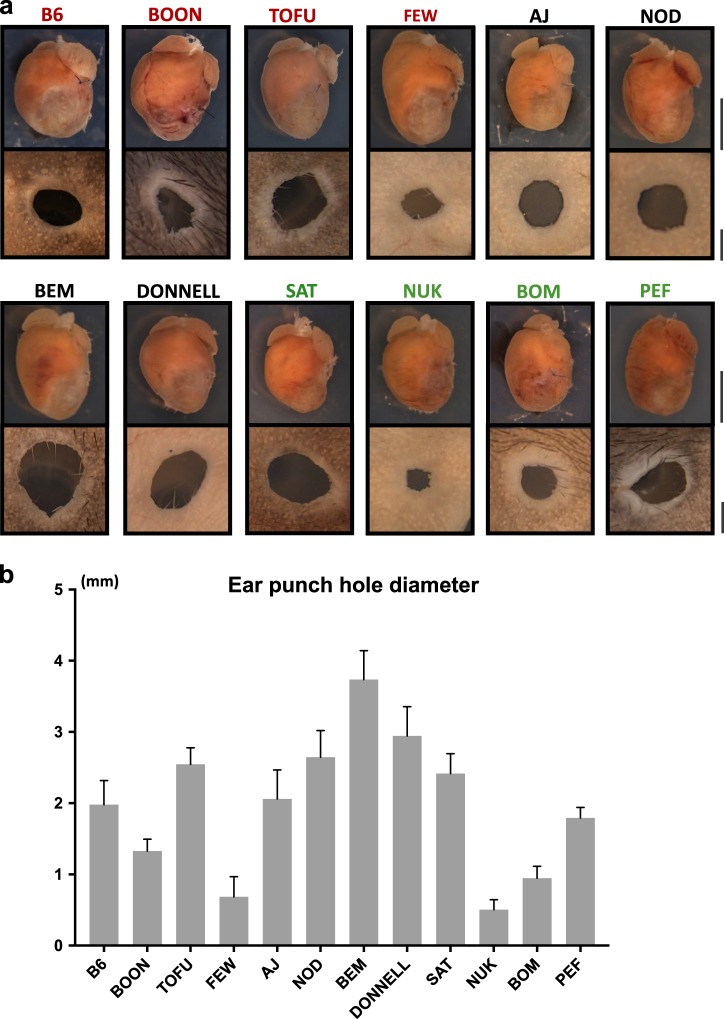
Comparative analysis of heart vs. ear regeneration. **a** Representative images of selected CC strains one month after MI and ear punch. In green, strains with minimal LV remodeling after MI, and in red, strains with adverse LV remodeling after MI. Note the poor correlation of the degree of ear punch healing with the outcome of MI. Scale bars, 5 mm (heart images) and 1 mm (ear punch images). **b** Quantification of the ear punch hole area. Data are presented as mean value ± SD of minimum three biological replicates

To compare the efficiency of heart repair with a recognized model of tissue regeneration established with the Murphy Roths Large (MRL/MpJ) mouse strain,^28^ ear punch closure analysis was performed on selected CC RI strains. Surprisingly, no direct correlation between these two regenerative scenarios was seen (Fig. 5). Specifically, the BEM_AG and PEF_EC strains recovered relatively well from MI response, yet were not as efficient in ear punch hole healing. By contrast, the BOON_HF and FEW_FD strains responded poorly to the MI challenge, yet healed ear punch holes effectively. These results support the presence of distinct genetic programs underlying cardiac vs. ear wound repair.

### Cardiac gene expression profiling and functional annotation

To highlight the molecular differences among CC hearts that might predict functional variation in in cardiac injury response, we performed systematic gene expression profiling on the uninjured hearts of selected CC RI strains. Principal component analysis (PCA) on whole-transcriptome profiles of 27 strains employed in this study did not detect any clustering across strains on the transcriptome-wide level that would correlate with the observed cardiac injury response (Supplementary Fig. S1). Strains with higher resilience in response to LVSD/HF did not cluster separately from more susceptible strains.

**Fig. 6:**
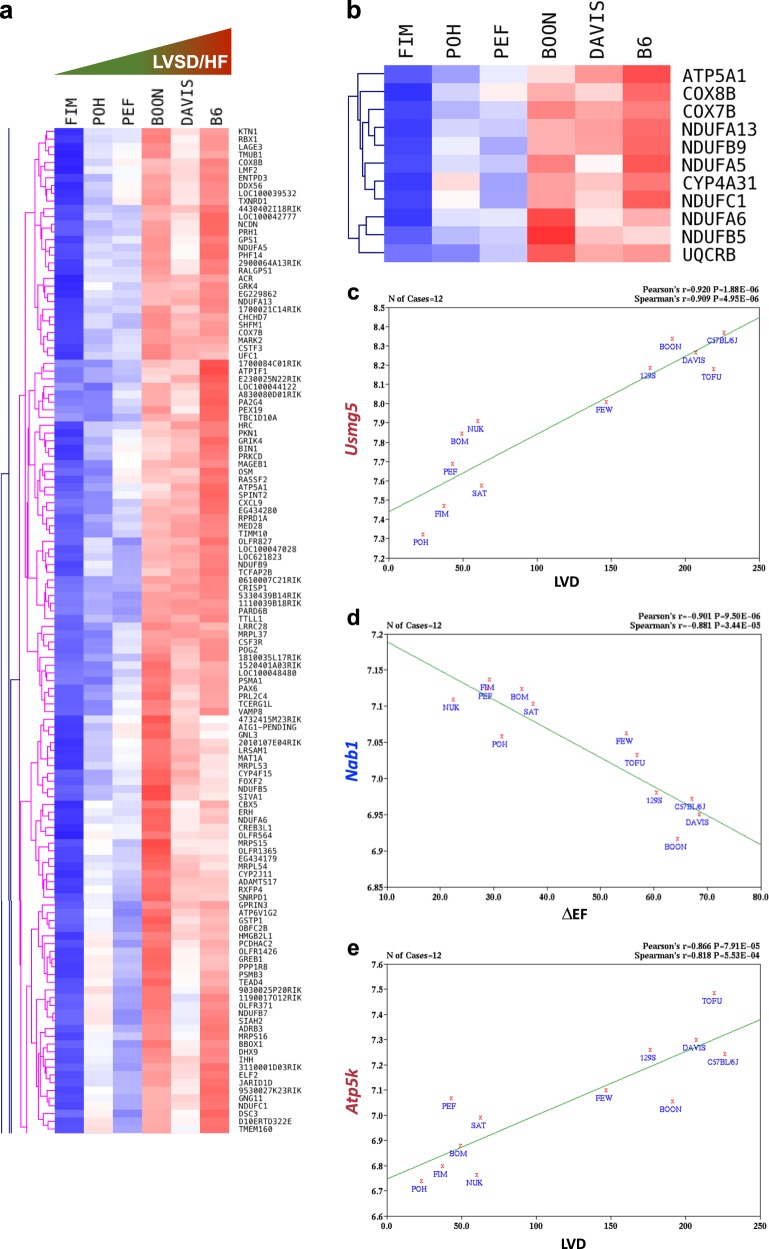
Expression profiling and functional annotation clustering. **a** A heatmap of a selected gene expression cluster correlating with MI-induced left ventricular systolic dysfunction (LVSD) and heart failure (HF) phenotype. The FIM, POH, and PEF strains displayed minimal LVSD, whereas the B6, DAVIS, and BOON strains displayed severe LVSD after MI (see Fig. 3A). **b** Expression profile of genes involved in oxidative phosphorylation overrepresented in cluster a (*p*-value > 2.00E − 04). **c**–**e** Representative correlation plots of top correlates of gene expression vs. LVSD/HF-related traits (LVD and ΔEF). **c** Positive correlation of *Usmg5* mRNA expression levels with the LVD phenotype (correlation coefficient − 0.92, *p*-value = 1.88E − 06). **d** Negative correlation of *Nab1* mRNA expression levels with the ΔEF phenotype (correlation coefficient − 0.90, *p*-value = 9.50E − 06). **e** Positive correlation of *Atp5k* mRNA expression levels with the LVD phenotype (correlation coefficient − 0.87, *p*-value = 7.91E − 05)

To detect meaningful variations in gene expression that could be indicative of predisposition to or protection from MI-induced LVSD/HF, hierarchical clustering on genes was performed in the 12 strains with most pronounced dissimilarities in the LVSD/HF phenotype (see Fig. 3a). No apparent clusters correlating with the phenotype could be identified, in agreement with the PCA results. However, when clustering was repeated on six strains from the extremities of the LVSD/HD spectrum (FIM_DF, POH_DC, PEF_EC, BOON_HF, DAVIS_BA, and B6; see Fig. 3a), five prominent clusters of gene expression profiles displayed correlation with LVSD/HF. Enrichment analysis using PANTHER^29^ and DAVID^30^ revealed one cluster (Fig. 6a) enriched in genes involved in oxidative phosphorylation (GO-Slim Biological Process GO:0006119, corrected *p*-value = 2.02E − 04 and KEGG (Kyoto Encyclopedia of Genes and Genomes) pathway mmu00190, corrected *p*-value = 2.33E − 05). A heatmap of the genes from PANTHER and DAVID enrichment lists (*Atp5a1*, *Ndufa5, Ndufa6*, *Ndufb5*, *Ndufb9*, *Ndufa13*, *Ndufc1*, *Cox8b*, *Cox7b*, *Cyp4a31*, *Uqcrb* (Fig. 6b)) evidenced reduced gene expression across the strains with minimal LV remodeling and elevated gene expression in the strains with distinct LVSD.

To validate this finding and to explore the connections between individual gene expression levels and predicted LVSD/HF phenotypes, GeneNetwork/WebQTL correlation analysis was performed between the cardiac expression dataset and the LVD and ΔEF traits using 12 strains most susceptible (B6, DAVIS_BA, BOON_HF, TOFU_FB, 129 × 1, FEW_FD) and resilient (FIM_DF, POH_DC, PEF_EC, BOM_GB, NUK_AC, SAT_GA) to LVSD/HF (see Fig. 3a). Of the 500 top candidate genes with a correlation coefficient above ± 0.60, 343 were common for both traits. Enrichment tests on these 343 correlates revealed the “generation of precursor metabolites and energy” (GO:0006091) and “oxidative phosphorylation” (mmu00190) terms being overrepresented (corrected *p*-values = 4.35E − 02 and 1.49E − 03, respectively) comprising *Atp5a1*, *Atp5e*, *Atp5k*, *Cox7b*, *Ndufb4*, *Ndufb5*, *Ndufb9*, *Sdhd*, and *Uqcrb*. All these genes displayed positive correlations between messenger RNA expression and adverse MI outcome. *Usmg5* (also known as *Atp5md*; ATP synthase membrane subunit DAPIT) was one of the top scoring correlates on the list (Fig. 6c) and had expression also positively correlating with the LVSD/HF phenotype. Usmg5 co-purifies with mitochondrial ATP synthase^31^ and is critical for maintaining its function.^32^ Collectively, these data support a plausible connection between elevated intrinsic levels of oxidative phosphorylation and predisposition to LVSD/HF. Notably, the top negative correlate *Nab1* (Ngfi-A-binding protein 1; Fig. 6d) is a transcriptional repressor described as a negative regulator of pathological cardiac hypertrophy.^33^

A summary of the 50 top correlates common for LVD and Δ%EF traits is presented in Table S3 (Supplementary Material). In addition, two other genes with expression positively correlating with the LVSD/HF phenotype are worth noting: (1) *Traf3* (tumor necrosis factor receptor-associated factor 3), a major regulator of the innate immune response with expression levels associated with neurological and cardiovascular diseases;^34^ and (2) *Cxcl9*, a chemokine associated with various pathological conditions including dilated cardiomyopathy, HF, arrhythmias, and heart transplant rejection.^35^

### Quantitative trait locus analysis

**Fig. 7:**
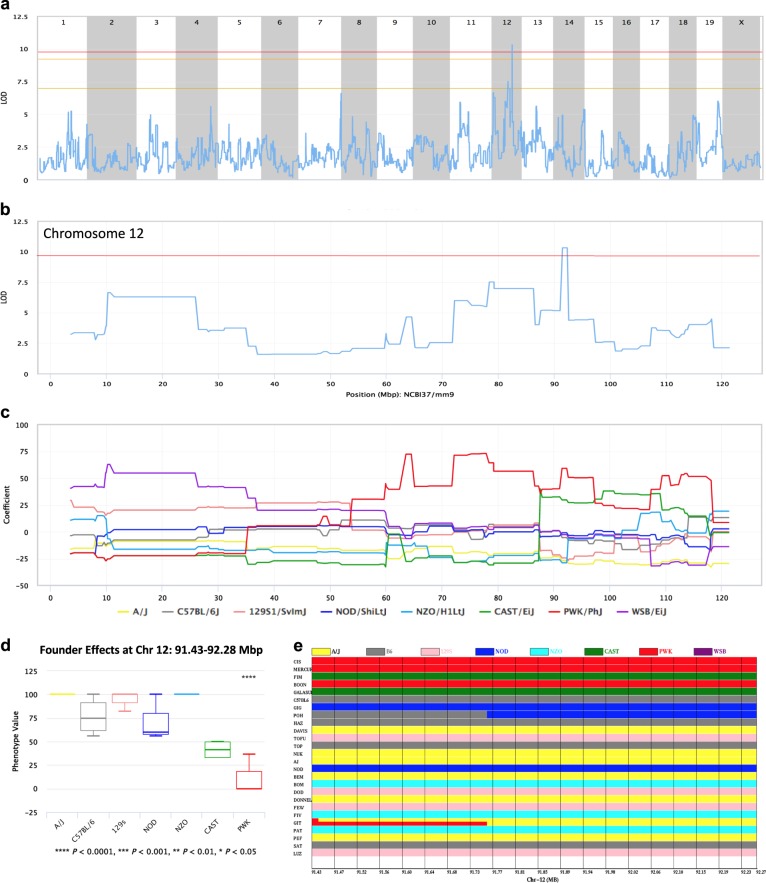
QTL mapping of the myocardial rupture trait. **a** A QTL map from a genome-wide scan comparing CC strains according to the myocardial rupture trait value. Chromosomal positions are presented on the *X* axis, whereas the *Y* axis shows LOD scores. The three significance threshold lines (from bottom to top) correspond to genome-wide permutation *P*-values < 0.63 (suggestive) < 0.10 (approaching significance) and ≤ 0.05 (significant, in red). **b** A QTL map of chromosome 12 containing the locus with the greatest LOD score for this trait. **c** A founder coefficient plot showing the contribution of the eight CC founder alleles at each position along chromosome 12. Note that the chromosomal positions in parts B and C line up, allowing direct comparison. **d** At this QTL, the PWK founder strain has a significant effect on the trait. **e** A haplotype figure showing the founder strain haplotype present in each CC strain at the peak QTL region. Note that three of the five CC strains with the lowest values for this trait (at the top of the haplotype stack) have the PWK haplotype at this position, whereas two of these five strains have the CAST haplotype. Genomic coordinates are relative to the NCBI37/mm9 mouse genome assembly

Quantitative trait locus (QTL) analysis of variation in MI response revealed one significant QTL (*P* ≤ 0.05) on Chr 12 (logarithm of the odds (LODs) = 10.3) for susceptibility to myocardial rupture (survival) (Fig. 7a, b). This QTL spans the region 91.430–92.280 Mbp. At this region, the PWK/PhJ and CAST/EiJ founder strains have predominant influences on the trait, with the effects of the PWK strain being significant (*P* < 0.0001; indicated by the red and green intervals in Fig. 7c, respectively; the founder effects summary in Fig. 7d and the haplotype plots sorted by phenotypic effect, Fig. 7e). The − 2 LOD drop region includes only three protein coding genes: *Nrxn3*, *Dio2*, and *Cep128*. However, all missense single nucleotide polymorphisms (SNPs) in this region are contained within *Cep128*. This gene encodes a centriole appendage protein negatively regulating ciliogenesis^36^ that has a central role in pathogenesis of human congenital heart disease.^37^ Of the nine missense SNPs in *Cep128*, four were unique for the PWK haplotype: rs261503899 (C > T), rs46662645 (A > T), rs262007891 (G > C), and rs266165675 (C > T) (*p*-value 1.48e − 07); and four to both the PWK and CAST haplotypes: rs252980304 (C > T), rs48026157 (A > C), rs51557552 (T > C), and rs46446778 (T > C) (*P* = 1.06e − 05). This indicates that *Cep128* is an excellent candidate for the causative gene for cardiac rupture at this locus, with the abovementioned SNPs mediating the myocardial rupture trait.

**Fig. 8:**
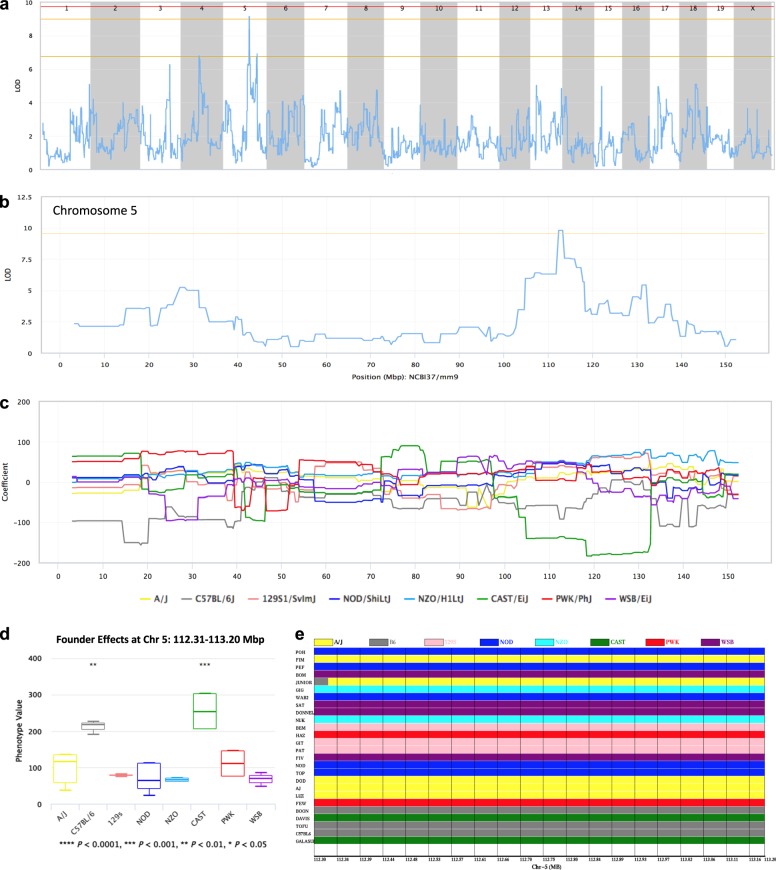
QTL mapping of the left ventricular dilation (LVD) trait. **a** A QTL map from a genome-wide scan comparing CC strains according to the LVD trait value. See Fig. 7 for other details. **b** A QTL map of chromosome 5 containing the peak locus that is above the line indicating LOD scores approaching significance. **c** A founder coefficient plot for chromosome 5 showing the contribution of the eight CC founder alleles at each position along the chromosome. At the peak QTL region, the B6 and CAST founder strains have the most effect on the trait (positive contribution). **d** Founder effects for chromosome 5 at the position of the greatest LOD score, showing significant contributions by the CAST and B6 founder strains. **e** A haplotype figure showing founder strain haplotype present in each CC strain at the peak QTL region on chromosome 5. Note that three of the five CC strains with the greatest values for this trait (at the bottom of the haplotype stack) have the B6 haplotype at this position, whereas two of these five strains have the CAST haplotype. Genomic coordinates are relative to the NCBI37/mm9 mouse genome assembly

Another QTL approaching significance (*P* ≤ 0.10) was associated with the LVD trait (Fig. 8a, b). This QTL is located on Chr 5 (LOD = 9.1) and falls into the region 112,310–113,205 Mbp containing multiple genes (*Cryba4*, *Tpst2*, *Tfip1*, *Srrd*, *Gm6583*, *Hps4*, *Sez6l*, *Asphd2*, *Gm6588*, and *Myo18b*). Both the CAST/EiJ and C57BL/6J parental strains displayed significant effects with this QTL (Fig. 8c–e), suggesting that both strains harbor SNP(s) that mediate predisposition to LVD (*p*-value 9.64e − 04). Although the majority of significantly associated SNPs in the Chr 5 QTL region were specific to the CAST haplotype, nine SNPs were present both in CAST and B6, specifically rs33218387 (G > A), rs253490750 (C > T), rs228327059 (C > T), rs32116976 (C > T), rs32122208 (G > A), rs32118052 (G > C) and rs32118955 (C > T), and rs47968507 (G > C) and rs46076017 (T > C). These SNPs would be the best candidates to explain the QTL.

Remarkably, 24 of the missense SNPs significantly associated with this QTL reside in *Myo18b*, with two of these (rs47968507 (G/C) and rs46076017 (T/C)) shared by both B6 and CAST. *Myo18b* has been implicated in sarcomere assembly and maintenance of myofibril structure^38^ as well as human skeletal and cardiomyopathies when mutated.^39,40^ In addition, a polymorphism in *Sez6l* has been associated with ischemic heart disease in humans^41^ and five SNPs in *Sez6l* are significantly associated with the LVD trait.

A full list of SNPs associated with the two QTL, including their precise genomic location and predicted effects on protein function, is presented in Tables S4 and S5 (Supplementary Material). All other traits analyzed (e.g., BWt, LV mass, heart rate, EF, and EF change (%EF and Δ%EF), LV SV, LV wall thickness) did not produce QTL with a *P* < 0.10, although some did have a suggestive *P* < 0.63.

## Discussion

It is widely recognized that most common human pathologies such as heart disease are genetically complex, requiring analysis of many thousands of individuals to achieve adequate statistical power for effective genetic dissection. Inbred mice are ideal model organisms for systems genetic analysis, but have been only modestly successful in mirroring human traits due to their limited genetic diversity. Key limitations in complex genetic mapping using inbred mice include low resolution of linkage approaches, a high degree of false positive signals found in murine association mapping (itself due to higher linkage disequilibrium among the common laboratory strains),^42,43^ and the critical need for permanent resources to apply systems-based approaches.

The genetically diverse CC mouse panel deployed here redresses these shortcomings and has already been successfully exploited to map genetic modifiers of many traits, including susceptibility to infection,^15–17^ drug toxicity,^21,44^ motor performance,^13^ hematological parameters,^14^ immunological conditions,^18,19^ reproduction,^20^ glycome repertoire,^22^ and traits associated with skin cancer.^23^ By employing the gold standard model of experimental MI to recapitulate the outcomes of human heart attack, the current study provides important insights into the genetic variation in cardiac function and disease. It discloses distinct genetic programs underlying specific aspects of cardiac response, underscores the diversity of regenerative strategies in different tissue types, and reveals novel factors and candidate genes that can now be experimentally validated.

Differential susceptibility of common laboratory mouse strains to myocardial rupture has been previously described.^45,46^ However, these studies were performed with a very limited number of strains and no molecular determinants were identified. Increased susceptibility of the B6 and 129 strains to myocardial rupture was noted, with 129 substrains being the most affected (notably previous reports used 129s6 and 129sv^45,46^).

The mapping power of genetic studies in mice has been limited by the lack of high throughput experimental challenges that accurately recapitulate human MI. Nevertheless, two significant QTL were detected here that warrant functional validation. SNPs in the *Cep128* gene corresponding to the rupture-associated QTL on Chr 12 suggest a possible molecular determinant of myocardial wall strength, as the role of cilia in heart development and congenital heart disease is well established.^37,47,48^ Primary cilia and ciliogenesis are also required for cell migration essential for organogenesis and tissue repair.^49,50^ Destabilization of the actin cytoskeleton has been recognized as a dominant inducer of ciliogenesis^51^ and roles of cilia in mechanotransduction are reviewed in ref. ^52^. These considerations strongly implicate cilia-related signaling in maintenance of LV integrity during the inflammatory phase following MI that involves active actin undefined and cell migration. Although *Cep128* was not detected in the recessive forward genetic screen that implicated several genes related to ciliogenesis and cilia-mediated signal transduction in pathogenesis of congenital heart disorders,^37^ it is nevertheless highly plausible that SNPs in *Cep128* affect maintenance of LV myocardium integrity and repair during the highly migratory inflammation phase post MI.

Of the significant SNPs in the Chr 5 QTL region, missense variants in *Myo18b* are the most likely candidates for predisposition to cardiac dilation, considering the widely acknowledged role of *Myo18b* in myofibril assembly and myopathies.^38–40,53^ However, none of these are unique to CAST and B6—the only two strains displaying significant founder effects for the LVD trait (see Table S5). Therefore, it could be possible that a non-missense variant that is in common for CAST and B6 but not present in any other founder strains is the influential SNP for this trait. Alternatively, the same trait in CAST and B6 could be due to different SNPs in each strain. Moreover, the presence of the same SNPs in strains other than CAST and B6 (e.g., rs47968507 (G/C) and rs46076017 (T/C); Table S4) may be negated by other genomic contributions. Polymorphisms in *Sez6l*, another gene within the QTL on Chr 5, could be also contributing to the trait. In humans, the *SEZ6L* polymorphism rs663048 has been associated with ischemic heart disease and arterial hypertension.^41^ This specific SNP is not recapitulated in the CC mouse population; however, the region is highly conserved, and several other SNPs may influence the LVD phenotype (e.g., rs32116976, rs32122208, rs32118052, and rs32118955) (see Table S4). Indeed, it is possible that the concerted action of multiple SNPs may contribute to compromised function or levels of Myo18b and/or Sez6l protein, resulting in predisposition to HF.

Our analyses focussed on missense and splice variants within the − 2 LOD drop for both QTL, as no relevant nonsense, frameshift, or insertion/deletion variants were detected. However, we cannot rule out potential contribution of synonymous and/or non-coding SNPs. Differences in gene expression levels (more likely to be influenced by non-coding genetic variants) and transcript stability or translation efficiency due to synonymous changes,^54^ rather than changes in amino acid sequence of the resulting protein, could conceivably influence the phenotypes we measured. Moreover, the length of a microsatellite within an intron of *Myo18b* correlates with LVD (Supplementary Fig. S2), so this too may exert an effect, as intronic microsatellite instability had been previously associated with disease states (reviewed in ref. ^55^). Although neither *Cep128* nor *Myo18b* were represented on the gene expression array employed in this study, the results of quantitative PCR on complementary DNA from a selected set of CC RI strains using specific TaqMan gene expression assays revealed no correlation between the polymorphisms in these genes and the mRNA expression levels (Supplementary Fig. S3). This implies that the polymorphisms in these genes may result in a compromised function of Myo18 and Cep128 proteins rather than in alteration of mRNA expression and/or stability.

As HF is a disease of bioenergetic imbalance, it is interesting that several components of the oxidative phosphorylation machinery were upregulated in strains with adverse MI outcome. The heart is the greatest oxygen-consuming organ in the body, with no excess capacity for ATP production vs. utilization. Energy supply in the form of ATP is mandatory to sustain cardiac contractile and relaxation functions, and 90% of this requirement is met by mitochondrial oxidative phosphorylation that is finely adjusted to energy needs.^56^ Mitochondrial dysfunction has a key role in progression to HF, as evidenced by decreased ATP production consistently observed in various models of HF (reviewed in refs. ^56,57^) Thus, hearts with a higher intrinsic metabolic state and high energy consumption due to increased components of oxidative phosphorylation machinery (e.g., mitochondrial ATP synthases and ubiquinone oxidoreductase subunits) may be more promptly compromised after acute MI stress. Surpassing cardiac compensatory capacity results in suppression of ATP production and progression to HF. Intrinsic oxidative phosphorylation status and mitochondrial metabolic state could therefore serve as a biomarker for long term MI outcome prognosis, and as such represents an important target for therapeutic intervention to improve cardiac function.^58^

Other notable variables in transcript analysis of CC hearts include the following: (a) *Nab1*, a zinc finger transcription factor expressed in mouse cardiac myocytes, endothelial cells, and fibroblasts.^33^ *Nab1* is a negative regulator of pathological cardiac hypertrophy, as *Nab1*-overexpressing mice are resistant to ɑ- and β-adrenergic stimulation ventricular hypertrophy;^33^ (b) *Traf3*, a member of the TRAF adaptor protein family and a major regulator of the innate immune response, which has been identified as a positive regulator of cardiac hypertrophy in response to high pressure levels both in mouse and human hearts;^34,59^ and (c) *Cxcl9*, a circulating chemokine ligand of the CXCR3 receptor and T-cell chemoattractant, reported to be a valid biomarker for the development of LV dysfunction and HF.^35,60^ Thus, in homeostatic conditions, cardiac *Nab1*, *Traf3*, and *Cxcl9* levels may be predictive of adverse LV remodeling in response to MI.

Given the importance of infarct scar size and properties to LV function,^61^ it is interesting that features of infarct scar structure quantified in this study did not correlate with post-infarction dilation or changes in function in the subset of animals where this was assessed. Collagen AF was fairly similar across the strains analyzed and scars with lower AF tended to be thicker, suggesting the total amount of collagen produced in each strain was remarkably similar. Although the alignment of the collagen fibers varied widely across the strains, there was no clear relationship between collagen alignment and the development of failure.

Recently, a correlation was reported between mononuclear cardiomyocyte frequency, CM proliferation, post-MI outcome, and expression of *Tnni3k* across strains of the Hybrid Mouse Diversity Panel.^62^ However, *Tnni3k* genetic ablation had no effect on cardiac function or response to injury,^62^ and in the current study no correlation was observed between MI-induced HF phenotype in CC RI strains and *Tnni3k* locus polymorphisms or mRNA expression levels (Supplementary Fig. S2). Therefore, it is likely to be that this genetic locus operates in concert with other modifiers to influence CM proliferation and predisposition to heart disease.

The results of this pilot study demonstrate the power of genetic mapping in the identification of new modifiers in heart disease and underscore the added value of establishing mouse models that more accurately reflect the variation in human pathology. Large-scale human genome sequencing has empowered gene association studies and promises to catalyze a transformation of medicine. Deeper knowledge of human sequence variation and application of systems approaches has already begun to enrich our understanding of the diversity in disease across the human population.^63^ The pace of translating human genome research, and functionalizing these correlations for the practice of healthcare will now be heavily reliant on the use of appropriate organismal models that accurately reflect the human condition, such as the CC used here. From a clinical perspective, the development of predictive biomarker panels common to mouse and man would allow susceptibilities to be detected and heart disease to be treated much earlier than is currently possible. Such panels would provide a rapid screen for cardiovascular disease susceptibility and early-onset pathology, and could improve disease management by providing markers for risk, early detection, prognosis, and therapeutic response that would be immediately applicable to pharmacological screening.

## Materials and methods

### Animal handling

All mouse experiments were performed at Monash University and The University of Western Australia and were approved by the Animal Ethics Committees from the respective university. The CC mouse lines were bred and supplied by Geniad Pty Ltd (Perth, WA, Australia)^64^ and housed at the Animal Resources Centre (Perth, WA, Australia). At 4–5 weeks of age, mice of backcross generation > 25 were shipped to Monash Animal Services where they were housed in temperature-controlled facilities on a 12 h light/dark cycle with chow and water supplied ad libitum. Experiments were performed on 12-week-old male mice, unless stated differently.

The only four CC founder strains available within Australia (A/JArc, C57BL/6JArc, NOD/ShiLtJArc, and 129 × 1/SvJArc) were sourced from the Animal Resources Centre (Perth, WA, Australia). The 129 × 1/SvJArc substrain was used instead of 129S1/SvImJ, and therefore this strain was excluded from the QTL analysis.

Selected RI strains in the CC panel have been maintained as independent colonies for many generations in different international locations and are therefore susceptible to genetic drift. The RI strains analyzed in the current study were generated and maintained at the Animal Resources Centre (ARC)/Geniad Pty Ltd (Western Australia), and therefore the ARC/Geniad nomenclature has been retained to indicate their specific origin. See Table S6 (Supplementary Material) for corresponding nomenclature of the CC strains maintained at The Jackson Laboratory and Table S7 (Supplementary Material) for abbreviations of the mouse strains employed in this publication.

### Ultrasound analysis

Cardiac morphological and functional parameters were assessed with a Vevo2100 ultrasound imaging system (FUJIFILM/VisualSonics Inc., Canada) with an ultra-high frequency linear array transducer (VisualSonics MS400, 18–38 MHz). Mice were anesthetized with 4% isoflurane in oxygen and placed in a supine position on a temperature-controlled imaging platform under continuous supply of 1.5% isoflurane. Chest hair was removed using a hair removal cream (Nair) and ultrasound transmission gel (Aquasonic, Parker Laboratories, USA) was applied on the thorax. The heart rate was continuously monitored and maintained at 400–450 bpm. Measurements and calculations were performed using VevoLAB (v1.7) software package (FUJIFILM/VisualSonics), presented in detail in Supplementary Material.

LVD parameter was calculated as % increase in LV volume after MI (LVD = LV-volume;d-afterMI/LV-volume;d-beforeMI * 100 − 100). ΔEF was calculated as % change in EF (ΔEF = (EF-beforeMI − EF-afterMI)/EF-afterMI * 100). Results are provided as a mean ± SD of the mean for 4–10 mice from each CC strain. Data were analyzed using GraphPad Prism (v7) software (IBM, Armonk, NY, USA).

### Coronary artery ligation model of MI

The LCA was permanently ligated to model MI. Mice were anesthetized with 4% v/v isoflurane, attached to an artificial respirator (SV of 200 µl at 120 strokes per min) via endotracheal cannulation and maintained under 2% v/v isoflurane anesthesia through the surgery. An incision was made through the muscle between the fourth and fifth intercostal space to access the heart. A 6 mm tapered point needle with an 8-0 polyethylene suture was passed through the myocardium underneath the LCA approximately 1 mm below the tip of the left auricle. The ligature was permanently tied around the LCA with three knots. The mouse thoracic cavity was then closed and sutured. A 0.05 µg/g buprenorphine solution was administered by subcutaneous injection twice daily for 3 consecutive days post surgery. Mice were allowed to recover for 1 month and during this time general condition and BWt were assessed at regular intervals. Only mice with > 30% of LV ischemia were used for further analysis.

### Specimen collection and histology

At experimental endpoint, mice were killed by CO_2_ asphyxiation and perfused with phosphate buffered saline (PBS) to remove blood. Hearts were excised, cleaned, fixed in 4% paraformaldehyde overnight and washed in PBS. Imaging of whole-mount hearts was carried out using an Olympus SZX16 stereo microscope equipped with an Olympus DP70 camera (Olympus, Japan) and running analySIS LS Starter v2.6 Software (Olympus Soft Imaging Solutions, Germany). Samples were further dehydrated and kept in 80% v/v ethanol at 4 °C for further experiments.

### Scar morphology and quality analysis

Formalin-fixed hearts were divided into three transverse rings; the middle ring, encompassing the region halfway between the base and the apex, was used for histologic analysis. Scar samples extending from the anterior RV insertion through a 90° arc toward the lateral well were cut from the middle ring, dehydrated, transferred to paraffin, and embedded. Serial sections of 7 µm thickness oriented parallel to the epicardial surface were cut with a microtome (Leica) beginning at the epicardium and progressing to the endocardium. The apparent wall thickness was calculated based on the difference between the first and last sections that included tissue in the center of the section. Sections representing the LV midwall (relative depth between 40–60% of the total wall thickness) were stained with picrosirius red for collagen and analyzed as described previously.^27^ An area of approximately 1 mm^2^ (1.13 × 0.85 mm) in the center of each midwall section was imaged under circularly polarized light (collagen fibers appear bright) and under brightfield illumination with a blue filter (collagen fibers appear dark). These two images were subtracted to isolate the collagen fibers, and the collagen AF and orientation were quantified by analyzing the subtracted image with custom software (MatFiber, http://bme.virginia.edu/holmes/downloads/index.html). Data were analyzed with circular statistics, generating a mean vector for each sample, indicating the average orientation (mean angle) and strength of alignment (MVL; 0 for random and 1 for maximally aligned) of the collagen fibers. Collagen alignment was considered statistically significant for a given strain if the values MVL_i_ * cos(2 * *θ*_i _– 2*θ_m_) were statistically different from 0 by a one-sample *t*-test, where MVL_i_, *θ*_i_ are the length and orientation of the individual sample vectors, and *θ*_m_ is the orientation of the group mean vector.^27^

### Cardiac gene expression analysis

Analysis of gene expression from the hearts of 55 CC strains was previously described in ref. ^65^, with 27 of these strains being those analyzed in the present study. Heart muscles were dissected immediately post-killing from 6-week-old, untreated CC male mice (*n* ≥ 3 per strain), snap frozen in liquid nitrogen, and stored at −80 °C until processing. An RNAeasy Fibrous Tissue kit (Qiagen) was used to extract RNA, with RNA quality determined with an Agilent Bioanalyzer. An RNA integrity number of 7 used as the minimum cut-off value.^66^ Based on the amount of RNA, samples from each mouse of the same strain were evenly pooled.

The MouseRef-8 v2 BeadChip (Illumina, USA) was used to expression profile 25,698 transcripts from the murine genome. A Bead Array Reader was used to scan the BeadChip, with raw data exported using the Illumina Genome Studio software (v2011.1). Using the lumi package in R (from the Bioconductor software suite (www.bioconductor.org), results were log2-transformed and normalized with Robust Spline Normalization. All data have been made publically available at http://130.95.9.22/webqtl.html.

### Quantitative real-time PCR

RNA samples from CC RI and founder strains were quantified using the Nanodrop (Thermo) and normalized for concentration for cDNA synthesis. cDNA synthesis was performed using Superscript IV VILO Reverse Transcription Kit (Thermo), as per the manufacturer’s instructions. TaqMan gene expression assays (Thermo) were used for quantification of mRNA levels of *Myo18b* (Mm01201863_m1), *Cep128* (Mm00725257_m1), and *Tnni3k* (Mm00621993_m1) genes with *Gapdh* used as an endogenous control (4352932E). Data were generated with the ViiA7 Real-Time PCR System (Applied Biosystems). Samples were run in technical triplicates, each group had two to five biological replicates. Relative quantification analysis was performed using the ∆∆CT method.

### Gene expression analysis, functional annotation, and trait correlation

To assess expression data quality and sample grouping, PCA for all tested strains was performed based on normalized cardiac expression data from the MouseRef-8 v2 BeadChip (Illumina, USA) platform. PCA and pre-processing of cardiac expression data were performed using the lumi package in R available via the Bioconductor software suite (www.bioconductor.org).

Heatmaps of the heart muscle gene expression dataset were generated by performing hierarchical clustering using Multi Experiment Viewer software (MEV v4.8.1 TIGR) with Euclidean Distance metric and default parameters. The PANTHER (v12.0) classification system (http://pantherdb.org/)^29^ was employed for statistical overrepresentation analysis using GO-Slim Biological Process annotation dataset with default parameters. The DAVID (v6.8) functional annotation tool (https://david.ncifcrf.gov/)^30^ was used for functional annotation clustering against the GOTERM_BP_Direct and KEGG_Pathway Databases. Resulting functional annotation clusters were corrected for multiple testing by Bonferroni adjustment. Clusters with corrected and *p*-values of *p* < 0.05 were considered significant.

The GeneNetwork^67^ (http://130.95.9.22/webqtl.html) was used for correlation analyses between the cardiac expression dataset (with gene expression threshold set to >7) and the quantitative traits. For the gene expression dataset, robust spline normalized data were transformed to produce *Z*-scores with a mean of 8 and a SD of 2 before entering into the GeneNetwork mouse gene expression database. This one unit difference in the transformed data was equivalent to a twofold difference in expression level.

### QTL mapping

Methods for mapping QTL using the CC strains have been described in detail and with examples (see, e.g., refs ^22,68,69^). In summary, each CC strain was genotyped using the 143,259 SNPs contained within the GigaMUGA (GeneSeek, Lincoln, NB) mouse genotyping array. The genotype of each CC strain was then compared with those of the eight CC founder strains, using a subset of SNPs. Specifically, 120,521 of the GigaMUGA SNPs were informative due to being reliably homozygous for each CC founder strain, yet with at least one founder having the non-reference allele. By applying the Hidden Markov Model algorithm in DOQTL, these informative SNPs were used to determine the most likely founder CC strain that had contributed the inferred haplotype at each chromosomal location for each CC strain. To conduct genome-wide association mapping the inferred haplotype probabilities were utilized by the GeneMiner online tool^64^ at http://130.95.9.124/Geniad2/ to interface with the R package QTLRel. A kinship matrix was used with a mixed model analysis to adjust for possible confounding due to cryptic relatedness between the CC strains as a random effect. Regression coefficients for the additive genetic effects of each one of the eight founder haplotypes were calculated at all genomic loci assessed. A likelihood ratio test was used for statistical analysis of the degree of association between loci and the specific trait in question.

Plots of the LODs scores were generated for each trait, with 1000 permutations used to determine significance thresholds. Threshold values were chosen at a genome-wide permutation *P*-value of ≤0.05 (significant), *P* < 0.10 (approaching significance), and *P* < 0.63 (suggestive). Two-LOD drop support intervals for each QTL were calculated and additional 10 kb intervals added to the boundaries to compensate for non-uniformity of SNP distribution. To compare the haplotype effect of the eight CC founder strains on each trait, coefficient plots were generated. Genome sequences from the Wellcome Trust Sanger Institute Mouse Genomes Project were used to identify candidate genes within each QTL region. The GeneMiner program was used to prioritize SNPs likely to alter protein structure and/or function that were within the QTL and present in the founder strain/s identified as conferring the trait being analyzed.

### SNP validation

Effects of identified SNPs were assessed using Variant Effect Predictor (VEP) software https://asia.ensembl.org/Tools/VEP (Ensembl release 93). VEP utilizes the SIFT (Sorting Intolerant From Tolerant) program to predict whether an amino acid substitution affects protein function.^70^ The SIFT score is the normalized probability that the amino acid change is tolerated. Substitutions with scores less than 0.05 are predicted to be deleterious.

The PCR was performed on mouse tail tip DNA, targeting selected genomic regions using Phusion® High-Fidelity DNA Polymerase (New England Biolabs). PCR fragments were purified using the NucleoSpin® Gel and PCR Clean-up kit (MACHEREY-NAGEL). Sanger sequencing was performed at the Micromon facility (Monash University). Sequencing alignment and SNP analysis were performed using the DNASTAR MegAlign tool (Lasergene® Core).

### Reporting summary

Further information on research design is available in the Nature Research Reporting Summary linked to this article.

## Supplementary information

Supplementary Information

Reporting Summary

## Data Availability

The microarray expression data have been made publicly available at the GeneNetwork http://130.95.9.22/webqtl.html. Other datasets that support the findings of this study are available from the corresponding authors upon reasonable request.

## Supplementary information

**Supplementary information** accompanies the paper on the *npj Regenerative Medicine* website (10.1038/s41536-019-0067-6).
