# Supplementary material for: Variable outcomes of human heart attack recapitulated in genetically diverse mice
Source: NPJ Regen Med. 2019 Mar 4;4:5. doi: 10.1038/s41536-019-0067-6 (PMC6399323; doi:10.1038/s41536-019-0067-6)
Supplement: Supplementary file 1 — Supplementary Information [file 41536_2019_67_MOESM1_ESM.pdf]

Table S1 Summary of morphological and functional parameters of selected CC strains based on echocardiographic images

12-week-old male mice in homeostasis

Strain / Parameter*	BWT (g)	LV Mass (mg)	ENDarea;d (mm2)	ENDarea;s (mm2)	EPIarea;d (mm2)	EPIarea;s (mm2)	Heart Length;d (mm)	Heart Length;s (mm)	FAC (%)	EF (%)	Stroke Volume (µL)	LV Volume;d (µL)	LV Volume;s (µL)	LVPW;d (mm)	LVPW;s (mm)	PA Peak Pressure (mm Hg)
129X1/SvJArc (10)	28.6 ± 1.7	95.17 ± 7.27	13.30 ± 0.93	7.41 ± 1.12	24.64 ± 1.34	20.23 ± 1.60	7.29 ± 0.18	6.56 ± 0.16	44.44 ± 5.02	51.91 ± 4.80	34.94 ± 2.75	67.60 ± 4.78	32.65 ± 5.14	0.70 ± 0.03	1.00 ± 0.04	2.34 ± 0.34
A/JArc (11)	24.1 ± 1.1	69.42 ± 8.47	9.62 ± 0.46	5.09 ± 0.36	18.90 ± 1.25	15.61 ±0.87	6.63 ± 0.19	6.06 ± 0.15	46.96 ± 4.39	53.29 ± 3.47	25.67 ± 2.04	48.20 ± 3.10	22.54 ± 2.47	0.65 ± 0.06	1.04 ± 0.08	2.32 ± 0.44
C57BL/6JArc (9)	26.3 ± 1.9	82.82 ± 7.05	13.13 ± 0.90	8.03 ± 0.92	22.30 ± 3.48	18.18 ±3.75	6.97 ± 0.11	6.41 ± 0.16	38.86 ± 6.31	47.33 ± 4.91	32.04 ± 3.09	67.82 ± 4.01	35.78 ± 4.40	0.68 ± 0.06	0.97 ± 0.08	2.00 ± 0.38
NOD/ShiLtJArc (11)	26.3 ±2.2	78.95 ± 11.10	11.34 ± 0.74	5.63 ± 0.84	21.31 ± 1.71	17.49 ±1.67	7.05 ± 0.19	6.25 ± 0.26	50.37 ± 6.51	57.53 ± 5.76	32.77 ± 3.84	56.91 ± 3.55	24.14 ± 3.63	0.73 ± 0.07	1.14 ± 0.10	2.63 ± 0.54
BEM_AG (12)	30.7 ± 3.3	88.85 ± 5.60	10.80 ± 1.15	5.38 ± 1.11	22.16 ± 1.76	18.52 ±1.42	6.99 ± 0.25	6.51 ± 0.17	50.57 ± 6.13	56.98 ± 6.05	30.99 ± 2.56	54.90 ± 6.71	23.91 ± 5.90	0.82 ± 0.06	1.42 ± 0.13	2.48 ± 0.49
BOM_GB (15)	23.1 ± 1.7	89.92 ± 9.01	11.57 ± 1.16	6.49 ± 0.90	23.81 ± 1.91	20.48 ±1.85	6.42 ± 0.23	5.89 ± 0.23	43.93 ± 5.69	51.03 ± 4.89	30.99 ± 3.86	54.90 ± 5.47	23.91 ± 3.77	0.80 ± 0.09	1.16 ± 0.11	2.97 ± 0.55
BOON_HF (10)	30.2 ± 2.0	98.57 ± 10.19	12.14 ± 0.88	7.27 ± 0.49	23.97 ± 1.36	20.11 ±0.99	7.36 ± 0.17	6.85 ± 0.19	39.90 ± 4.46	46.14 ± 3.61	31.34 ± 3.49	67.06 ± 5.65	35.72 ± 3.80	0.78 ± 0.07	1.14 ± 0.09	2.44 ± 0.33
CIS_AD (8)	24.9 ± 2.4	99.55 ± 9.10	11.23 ± 1.21	5.94 ± 1.05	23.19 ± 1.96	19.39 ±1.59	7.40 ± 0.31	6.69 ± 0.20	47.35 ± 5.44	55.03 ± 5.01	33.80 ± 4.64	61.54 ± 7.99	27.74 ± 5.24	0.87 ± 0.06	1.27 ± 0.11	2.37 ± 0.40
DAVIS_BA (6)	23.8 ± 0.9	93.59 ± 9.51	12.04 ± 0.49	7.73 ± 0.23	23.45 ± 0.71	19.54 ±1.26	7.29 ± 0.11	6.61 ± 0.15	35.68 ± 4.25	43.30 ± 5.05	29.44 ± 5.22	67.58 ± 4.73	38.14 ± 0.58	0.71 ± 0.08	1.07 ± 0.09	3.02 ± 0.40
DOD_AH (6)	32.0 ± 2.3	106.97 ± 8.96	12.28 ± 1.37	6.67 ± 0.64	24.79 ± 2.12	21.32 ±2.26	7.66 ± 0.19	7.12 ± 0.31	45.55 ± 1.40	52.16 ± 1.49	32.75 ± 5.31	59.21 ± 6.49	28.11 ± 2.50	0.97 ± 0.09	1.41 ± 0.13	1.97 ± 0.45
DONNELL_HA (7)	26.4 ± 2.1	74.51 ± 10.31	9.18 ± 0.78	5.49 ± 0.72	19.47 ± 1.53	17.10 ±1.43	6.37 ± 0.27	5.87 ± 0.19	40.05 ± 5.58	46.13 ± 6.33	20.52 ± 2.41	44.67 ± 3.01	24.15 ± 3.87	0.79 ± 0.13	1.24 ± 0.19	1.61 ± 0.49
FEW_FD (11)	24.8 ± 1.5	101.91 ± 7.96	11.32 ± 1.02	6.00 ± 0.93	23.79 ± 1.82	20.15 ±2.18	7.36 ± 0.22	6.82 ± 0.23	47.17 ± 4.81	53.13 ± 4.95	33.38 ± 3.54	63.14 ± 7.64	29.76 ± 6.09	0.98 ± 0.07	1.55 ± 0.15	2.56 ± 0.47
FIM_DF (11)	23.9 ± 2.5	73.15 ± 11.41	10.06 ± 1.71	6.45 ± 1.13	19.66 ± 3.05	17.04 ±2.38	6.75 ± 0.31	6.06 ± 0.27	35.70 ± 6.49	44.28 ± 6.57	22.94 ± 6.65	51.46 ± 9.85	28.52 ± 4.94	0.68 ± 0.09	0.98 ± 0.16	2.58 ± 1.09
FIV_AC (10)	25.8 ± 2.3	95.20 ± 7.56	9.98 ± 0.98	4.74 ± 0.54	22.07 ± 0.99	18.80 ± 1.24	6.95 ± 0.23	6.26 ± 0.24	58.88 ± 4.80	58.85 ± 4.73	29.17 ± 3.08	49.53 ± 2.83	20.36 ± 2.46	0.83 ± 0.06	1.34 ± 0.10	1.60 ± 0.32
GALASUPREME_CE (9)	28.1 ± 3.6	92.20 ± 11.20	9.92 ± 0.55	6.35 ± 0.19	20.80 ± 1.31	19.49 ±1.21	7.56 ± 0.16	7.18 ± 0.27	35.81 ± 3.98	41.05 ± 4.65	20.87 ± 3.42	50.70 ± 3.78	29.83 ± 2.25	0.87 ± 0.06	1.14 ± 0.10	1.70 ± 0.35
GIG_EF (13)	27.7 ± 1.8	81.62 ± 11.76	11.30 ± 1.12	5.88 ± 0.76	21.72 ± 2.00	18.18 ±1.71	6.92 ± 0.41	6.39 ± 0.36	48.03 ± 3.36	54.00 ± 2.95	31.47 ± 4.02	58.39 ± 7.47	26.92 ± 4.25	0.78 ± 0.06	1.25 ± 0.08	4.27 ± 1.57
GIT_GC (5)	33.1 ± 1.3	89.97 ± 6.97	12.27 ± 0.99	8.90 ± 1.21	24.12 ± 1.49	21.43 ±1.73	6.6 ± 0.19	6.34 ± 0.12	27.70 ± 4.83	32.67 ± 3.77	20.83 ± 1.86	64.15 ± 6.29	43.32 ± 6.07	0.73 ± 0.10	0.95 ± 0.012	2.56 ± 0.70
HAZ_FE (6)	19.4 ± 1.3	81.38 ± 8.00	11.90 ± 0.82	6.46 ± 0.46	22.24 ± 1.28	18.35 ±1.83	6.96 ± 0.18	6.34 ± 0.16	44.77 ± 1.67	49.49 ± 3.69	31.51 ± 2.38	63.85 ± 5.47	32.34 ± 4.68	0.68 ± 0.08	1.02 ± 0.015	3.41 ± 0.73
JUNIOR_GB (6)	23.2 ± 3.4	86.80 ± 11.59	9.67 ± 1.16	5.76 ± 0.90	20.41 ± 1.82	17.21 ±1.66	7.30 ± 0.47	6.69 ± 0.46	40.52 ± 5.77	47.22 ± 5.54	25.10 ± 3.09	53.53 ± 7.35	28.44 ± 5.95	0.84 ± 0.11	1.20 ± 0.16	2.24 ± 0.52
LUZ_FH (5)	23.1 ± 1.6	89.68 ± 7.61	11.32 ± 0.99	5.79 ± 0.55	23.27 ± 1.35	20.05 ±1.27	6.50 ± 0.10	5.76 ± 0.13	48.72 ± 4.59	57.17 ± 3.56	27.82 ± 1.60	48.71 ± 2.21	20.89 ± 2.37	0.76 ± 0.04	1.05 ± 0.07	3.15 ± 0.52
MERCURI_HF (5)	28.0 ± 3.5	92.71 ± 10.26	11.86 ± 0.96	7.16 ± 0.78	23.56 ± 1.33	20.50 ±0.75	7.00 ± 0.34	6.53 ± 0.44	39.68 ± 4.86	45.14 ± 4.59	27.44 ± 3.41	60.89 ± 5.63	33.45 ± 4.56	0.73 ± 0.04	1.02 ± 0.07	2.08 ± 0.45
NUK_AC (11)	32.4 ± 2.5	113.42 ± 7.98	12.58 ± 1.46	7.38 ± 1.15	26.32 ± 1.87	23.39 ±1.86	7.22 ± 0.42	6.75 ± 0.39	38.19 ± 6.87	46.22 ± 6.25	30.64 ± 5.85	66.27 ± 8.74	35.62 ± 6.05	0.87 ± 0.11	1.31 ± 0.14	1.86 ± 0.72
PAT_CD (6)	25.5 ± 1.2	102.58 ± 7.53	13.93 ± 0.97	8.30 ± 1.34	26.12 ± 1.12	22.21 ±1.15	7.42 ± 0.19	6.81 ± 0.21	40.58 ± 7.09	46.82 ± 7.41	34.83 ± 6.06	74.38 ± 6.47	39.55 ± 6.80	0.79 ± 0.12	1.15 ± 0.17	2.47 ± 0.74
PEF_EC (11)	30.3 ± 1.8	83.56 ± 6.40	9.77 ± 0.78	4.81 ± 0.26	21.49 ± 0.58	18.54 ±1.00	6.17 ± 0.30	5.54 ± 0.32	50.68 ± 2.51	59.37 ± 1.45	25.45 ± 2.03	42.88 ± 3.46	17.44 ± 1.65	0.92 ± 0.12	1.38 ± 0.16	2.83 ± 0.48
POH_DC (5)	27.5 ± 1.3	90.31 ± 10.86	9.75 ± 0.56	5.05 ± 0.14	21.45 ± 1.50	17.65 ±1.25	6.80 ± 0.26	6.41 ± 0.14	48.01 ± 4.35	52.52 ± 4.85	26.26 ± 5.66	49.65 ± 6.71	23.40 ± 2.18	0.75 ± 0.05	1.22 ± 0.12	2.66 ± 0.84
PUB_CD (6)	23.0 ± 2.2	64.39 ± 5.82	9.09 ± 0.90	5.47 ± 0.92	18.60 ± 1.44	15.68 ±1.65	5.92 ± 0.25	5.54 ± 0.26	40.04 ± 6.79	46.63 ± 6.37	16.86 ± 1.90	36.42 ± 3.96	19.56 ± 4.01	0.65 ± 0.07	0.95 ± 0.10	2.09 ± 0.73
SAT_GA (9)	28.7 ± 2.2	16.61 ± 4.08	11.06 ± 0.25	7.77 ± 0.64	23.23 ± 0.89	20.95 ± 0.61	36.60 ± 5.66	6.84 ± 0.46	36.86 ± 5.47	36.73 ± 5.56	19.20 ± 3.49	52.38 ± 6.72	33.18 ± 5.68	6.38 ± 0.24	0.82 ± 0.07	2.04 ± 0.33
TAS_FE (7)	18.7 ± 1.5	53.58 ± 5.75	8.98 ± 0.82	6.16 ± 0.65	16.42 ± 0.84	14.20 ±0.79	6.44 ± 0.26	6.01 ± 0.20	31.15 ± 6.30	38.36 ± 4.98	15.89 ± 3.05	41.96 ± 5.02	26.23 ± 2.91	0.56 ± 0.07	0.82 ± 0.10	1.34 ± 0.23
TOFU_FB (13)	25.4 ± 1.6	81.15 ± 4.97	9.03 ± 3.25	5.30 ± 0.74	20.07 ± 1.39	17.38 ±1.26	6.69 ± 0.13	6.33 ± 0.17	41.53 ± 4.41	47.44 ± 5.69	21.71 ± 1.32	46.16 ± 4.51	24.45 ± 4.86	0.85 ± 0.12	1.26 ± 0.20	3.14 ± 0.74
TOP_DA (8)	26.0 ± 1.9	98.73 ± 11.23	12.76 ± 0.86	7.20 ± 0.67	24.83 ± 2.00	21.17 ±1.54	7.02 ± 0.30	6.45 ± 0.27	43.55 ± 3.86	50.20 ± 4.61	32.69 ± 3.33	66.83 ± 4.48	33.25 ± 3.71	0.82 ± 0.11	1.22 ± 0.14	2.61 ± 0.64
WAB2_DH (8)	24.0 ± 1.0	81.70 ± 5.94	10.05 ± 0.54	6.34 ± 0.48	20.66 ± 0.87	17.80 ±0.89	6.82 ± 0.17	6.29 ± 0.18	36.89 ± 4.10	43.93 ± 3.07	22.16 ± 3.12	50.37 ± 5.15	28.22 ± 2.99	0.87 ± 0.06	1.18 ± 0.08	1.76 ± 0.45
YOX_DE (6)	26.4 ± 1.3	78.64 ± 4.63	12.91 ± 0.62	8.18 ± 0.55	23.09 ± 0.56	19.83 ±0.69	6.79 ± 0.19	6.14 ± 0.18	36.73 ± 1.96	44.23 ± 3.11	29.19 ± 2.60	65.94 ± 2.58	36.76 ± 2.26	0.67 ± 0.04	0.94 ± 0.06	2.92 ± 0.39

* Data is presented as mean value ± standard deviation (SD). Number of biological replicates is indicated in brackets next to the strain name.
Parameter definitions are provided below in "Ultrasound measurements and calculations" section.

Table S2 Summary of morphological and functional parameters of selected CC strains based on echocardiographic images
16-week-old male mice one month after MI

Strain / Parameter*	BWT (g)	AreaDist;d (mm2)	AreaDist;s (mm2)	AreaMid;d (mm2)	AreaMid;s (mm2)	AreaProx;d (mm2)	AreaProx;s (mm2)	Heart Length;d (mm)	Heart Length;s (mm)	EF (%)	LV Volume;d (µL)	LV Volume;s (µL)	Stroke Volume (µL)	PA Peak Pressure (mm Hg)	LVD (%)	DEF (%)
**129x1/SvJArc (3)**	31.3 ± 0.5	26.84 ± 5.81	24.76 ± 5.57	24.04 ± 4.13	21.03 ± 5.08	17.92 ± 1.22	13.57 ± 1.98	8.77 ± 0.89	8.15 ± 0.90	20.73 ± 6.98	188.38 ± 53.11	153.07 ± 53.99	35.31 ± 3.43	1.95 ± 0.70	175.23 ± 75.32	60.12 ± 8.90
**A/Jarc (8)**	26.4 ± 1.4	17.54 ± 4.74	16.13 ± 4.91	17.47 ± 4.67	14.38 ± 4.85	15.65 ± 3.15	10.80 ± 3.03	7.20 ± 0.47	6.75 ± 0.51	24.55 ± 5.99	112.73 ± 31.78	86.48 ± 29.46	26.25 ± 3.74	1.93 ± 0.28	134.05 ± 57.14	54.08 ± 9.27
**C57BL/6JArc (5)**	28.2 ± 2.2	30.16 ± 5.30	27.69 ± 4.78	30.22 ± 3.09	27.66 ± 3.04	25.93 ± 3.10	21.10 ± 2.65	8.10 ± 0.31	7.84 ± 0.39	14.34 ± 2.05	218.14 ± 33.60	186.87 ± 29.24	31.27 ± 6.84	1.59 ± 0.57	225.01 ± 50.18	70.17 ± 6.77
**NOD/ShiLtJArc (6)**	28.8 ± 1.0	14.51 ± 3.51	12.70 ± 4.15	18.20 ± 4.10	14.66 ± 4.78	18.04 ± 2.86	12.35 ± 3.31	7.72 ± 0.16	7.31 ± 0.25	27.50 ± 8.81	116.59 ± 21.35	86.08 ± 24.88	30.51 ± 4.91	2.26 ± 0.49	112.31 ± 43.62	51.99 ± 16.80
**BEM_AG (4)**	35.1 ± 5.0	11.71 ± 1.94	9.34 ± 2.18	15.35 ± 1.46	11.93 ± 1.48	14.41 ± 1.34	9.99 ± 1.83	6.94 ± 0.12	6.40 ± 0.21	30.51 ± 6.29	85.08 ± 10.04	59.37 ± 10.58	25.72 ± 3.99	1.76 ± 0.47	75.30 ± 39.27	42.2 ± 19.63
**BOM_GB (12)**	24.4 ± 1.5	13.39 ± 3.95	11.10 ± 4.02	15.34 ± 3.20	11.96 ± 3.26	15.80 ± 2.96	10.75 ± 2.97	6.54 ± 0.41	6.03 ± 0.48	31.28 ± 9.41	86.48 ± 21.56	60.84 ± 21.13	25.64 ± 4.25	2.05 ± 0.72	48.37 ± 27.10	34.96 ± 16.92
**BOON_HF (4)**	31.4 ± 5.1	28.58 ± 8.70	25.60 ± 9.83	29.17 ±13.01	27.01 ±14.49	20.44 ± 4.30	16.55 ± 5.99	8.37 ± 0.63	7.98 ± 0.36	17.95 ± 8.55	195.62 ± 86.27	165.26 ± 86.10	30.36 ± 4.88	1.88 ± 0.94	190.4 ± 123.11	64.13 ± 14.55
**DAVIS_BA (3)**	25.6 ± 0.14	27.92 ± 8.35	26.87 ± 8.39	30.63 ± 8.81	27.84 ±10.20	25.50 ± 4.61	20.63 ± 4.68	8.00 ± 0.66	7.68 ± 0.92	14.62 ± 7.42	215.61 ± 74.38	186.86 ± 79.52	28.75 ± 5.14	3.09 ± 1.65	206.45 ±103.76	68.16 ± 16.26
**DOD_AH (6)**	30.7 ± 2.7	17.89 ± 4.17	16.15 ± 4.53	18.67 ± 3.50	15.71 ± 3.35	16.68 ± 2.31	12.31 ± 1.70	7.95 ± 0.57	7.54 ± 0.38	21.48 ± 2.47	132.22 ± 32.44	104.02 ± 26.67	28.20 ± 6.67	1.76 ± 0.41	116.12 ± 55.65	58.76 ± 5.14
**DONNELL_HA (5)**	26.8 ± 2.6	10.90 ± 4.09	9.63 ± 4.92	12.71 ± 4.19	10.38 ± 4.19	11.82 ± 2.62	8.30 ± 2.03	6.68 ± 0.54	6.23 ± 0.61	26.62 ± 10.75	76.44 ± 29.75	57.95 ± 29.04	18.49 ± 4.21	1.03 ± 0.45	70.35 ± 58.89	40.79 ± 20.39
**FEW_FD (5)**	25.0 ± 2.7	21.02 ± 3.41	19.39 ± 3.37	21.08 ± 3.11	18.07 ± 3.65	18.73 ± 3.14	13.81 ± 3.67	8.03 ± 0.47	7.24 ± 0.58	24.21 ± 6.32	151.10 ± 24.33	115.65 ± 27.67	35.45 ± 4.64	2.55 ± 1.11	145.81 ± 63.82	54.50 ± 15.74
**FIM_DF (4)**	23.5 ± 2.0	9.62 ± 0.77	7.30 ± 1.58	12.31 ± 1.71	8.91 ± 2.20	13.42 ± 0.75	8.60 ± 1.52	6.86 ± 0.19	6.31 ± 0.23	35.79 ± 10.84	74.07 ± 5.31	47.37 ± 6.80	26.70 ± 9.14	2.63 ± 1.36	35.66 ± 19.76	28.84 ± 19.87
**FIV_AC (6)**	26.1 ± 1.8	12.47 ± 5.66	10.28 ± 5.58	12.96 ± 3.98	9.55 ± 4.24	12.94 ± 1.73	8.10 ± 1.85	7.25 ± 0.62	6.80 ± 0.59	32.74 ± 9.01	82.86 ± 24.25	57.18 ± 23.13	25.68 ± 3.93	1.64 ± 0.29	84.44 ± 42.59	50.56 ± 13.49
**GALASUPREME_CE (3)**	20.8 ± 2.7	28.00 ±10.26	25.78 ±11.95	22.63 ± 6.73	20.15 ± 9.12	19.12 ± 3.97	13.99 ± 3.84	8.71 ± 0.99	8.38 ± 1.14	19.35 ± 13.64	199.08 ± 78.37	167.26 ± 85.65	31.82 ± 8.20	1.86 ± 0.75	301.54 ±186.79	54.31 ± 31.02
**GIG_EF (3)**	27.6 ± 2.3	13.98 ± 3.78	12.29 ± 4.19	13.86 ± 2.78	10.67 ± 3.48	14.33 ± 1.18	8.51 ± 1.15	7.27 ± 0.36	6.79 ± 0.62	30.75 ± 5.51	96.42 ± 14.56	67.25 ± 15.03	29.17 ± 1.54	2.96 ± 1.00	61.91 ± 16.42	42.27 ± 9.42
**GIT_GC (3)**	33.0 ± 1.5	14.80 ± 0.83	13.14 ± 1.74	18.05 ± 3.24	15.08 ± 3.25	18.01 ± 1.59	12.29 ± 1.15	7.21 ± 0.32	6.81 ± 0.12	25.19 ± 3.19	113.04 ± 6.52	84.70 ± 8.00	28.35 ± 2.50	3.40 ± 0.94	79.23 ± 37.83	21.30 ± 14.98
**HAZ_EF (3)**	19.8 ± 1.2	15.59 ± 0.83	12.91 ± 1.83	18.07 ± 1.28	14.15 ± 1.65	18.48 ± 1.85	12.33 ± 1.92	7.51 ± 0.02	6.71 ± 0.27	32.41 ± 8.18	113.67 ± 6.80	76.87 ± 10.79	36.80 ± 8.85	3.27 ± 0.60	76.39 ± 14.86	35.69 ± 14.52
**JUNIOR_GB (3)**	24.8 ± 3.2	10.25 ± 0.83	8.42 ± 3.11	13.11 ± 4.21	10.56 ± 3.40	13.58 ± 1.83	9.50 ± 1.36	7.38 ± 0.09	7.07 ± 0.07	25.94 ± 3.94	87.37 ± 27.82	64.32 ± 18.97	23.05 ± 9.84	1.58 ± 0.30	57.63 ± 29.20	43.13 ± 16.38
**LUZ_FH (4)**	23.2 ± 0.5	18.65 ± 4.07	17.45 ± 4.53	19.22 ± 2.07	15.70 ± 2.20	17.40 ± 1.68	12.22 ± 1.49	7.08 ± 0.27	6.57 ± 0.27	23.74 ± 4.07	113.78 ± 12.21	86.87 ± 12.22	26.91 ± 4.77	2.00 ± 0.35	134.04 ± 23.99	44.86 ± 7.33
**NUK_AC (5)**	27.3 ± 4.1	12.87 ± 2.11	10.03 ± 2.66	15.37 ± 2.38	11.12 ± 2.55	14.94 ± 2.04	10.24 ± 1.81	7.12 ± 0.61	6.56 ± 0.58	35.15 ± 4.06	91.07 ± 14.44	66.77 ± 20.80	31.95 ± 6.80	2.42 ± 1.59	58.89 ± 33.18	22.02 ± 2.14
**PAT_CD (3)**	32.1 ± 4.1	15.73 ± 8.84	14.80 ± 8.60	18.76 ± 4.07	15.90 ± 2.42	16.97 ± 3.99	11.13 ± 1.93	7.65 ± 1.19	7.06 ± 1.03	24.25 ± 4.10	123.99 ± 50.22	92.73 ± 35.00	31.26 ±15.61	2.41 ± 0.52	81.38 ± 68.42	48.17 ± 0.89
**PEF_EC (6)**	27.2 ± 1.0	12.99 ± 4.07	11.21 ± 4.17	16.02 ± 3.69	11.82 ± 3.64	17.63 ± 3.46	10.57 ± 2.75	7.50 ± 0.42	7.02 ± 0.37	32.91 ± 8.81	102.85 ± 26.97	69.65 ± 22.75	33.19 ± 9.68	2.31 ± 0.42	41.61 ± 32.77	28.48 ± 18.97
**POH_DC (3)**	32.1 ± 2.4	8.01 ± 0.87	5.28 ± 0.71	10.97 ± 0.80	7.52 ± 0.33	11.77 ± 0.81	6.84 ± 0.35	6.05 ± 0.19	5.67 ± 0.15	40.24 ± 2.37	53.97 ± 6.71	32.23 ± 3.52	21.74 ± 3.58	1.64 ± 0.37	22.44 ± 17.68	31.18 ± 3.50
**SAT_GA (5)**	24.5 ± 2.5	10.47 ± 2.34	9.33 ± 2.66	12.31 ± 2.44	9.91 ± 2.65	12.95 ± 2.18	8.50 ± 1.42	6.35 ± 0.57	6.01 ± 0.61	28.73 ± 5.73	60.20 ± 10.62	49.34 ± 13.88	17.47 ± 3.21	2.34 ± 1.47	61.47 ± 34.68	37.75 ± 17.11
**TOFU_FB (10)**	19.8 ± 1.5	20.66 ± 2.44	19.63 ± 2.78	18.95 ± 2.85	17.53 ± 2.98	17.82 ± 1.95	12.98 ± 2.22	7.59 ± 0.35	7.28 ± 0.20	16.05 ± 5.12	134.51 ± 19.60	112.66 ± 16.38	21.85 ± 8.04	1.53 ± 0.39	217.55 ± 41.76	56.47 ± 17.69
**TOP_DA (4)**	27.5 ± 0.9	15.03 ± 6.30	12.71 ± 6.39	16.37 ± 4.19	13.78 ± 5.09	13.75 ± 5.25	9.58 ± 4.71	7.23 ± 0.68	6.77 ± 0.75	26.68 ± 8.31	99.07 ± 42.10	75.03 ± 41.26	24.04 ± 5.12	n/a**	112.18 ± 81.45	45.80 ± 16.95
**WAB2_DH (3)**	25.9 ± 2.2	14.42 ± 0.96	11.86 ± 1.07	18.13 ± 1.92	14.50 ± 2.42	17.30 ± 0.34	13.66 ± 1.95	7.28 ± 0.63	6.70 ± 0.64	25.96 ± 4.85	106.03 ± 11.25	78.52 ± 10.56	27.51 ± 5.14	2.21 ± 0.39	65.00 ± 23.99	44.52 ± 13.30

* Data is presented as mean value ± standard deviation (SD). Number of biological replicates is indicated in brackets next to the strain name.
Parameter definitions are provided below in “Ultrasound measurements and calculations” section.

** Not assessed

Ultrasound Measurement and Calculation definitions

Vevo2100 (FUJIFILM/VisualSonics)

Measurement definitions

Label	Description	Units	Generic type	Mode
Endocardial Area; d	Endocardial area in diastole	mm2	Area	B-Mode
Endocardial Major; d	Endocardial major in diastole	mm	Length	B-Mode
Endocardial Area; s	Endocardial area in systole	mm2	Area	B-Mode
Endocardial Major; s	Endocardial major in systole	mm	Length	B-Mode
Epicardial Area; d	Epicardial area in diastole	mm2	Area	B-Mode
Epicardial Major; d	Epicardial major in diastole	mm	Length	B-Mode
Epicardial Area; s	Epicardial area in systole	mm2	Area	B-Mode
Epicardial Major; s	Epicardial major in systole	mm	Length	B-Mode
LVPW;d	Left ventricular posterior wall (diastole)	mm	Depth	M-Mode
LVPW;s	Left ventricular posterior wall (systole)	mm	Depth	M-Mode

Calculations

Name	Description	Units	Formula
Endocardial Volume; d	Endocardial volume in diastole	μL	$\frac{4\pi}{2}\times\frac{End\ Major;d}{2}\times\left(\frac{End\ Area;d}{\pi\left(\frac{End\ Area;d}{2}\right)}\right)^2$
Endocardial Volume; s	Endocardial volume in systole	μL	$\frac{4\pi}{2}\times\frac{End\ Major;s}{2}\times\left(\frac{End\ Area;s}{\pi\left(\frac{End\ Area;s}{2}\right)}\right)^2$
Endocardial Stroke Volume (SV)	Stroke volume (SV)	μL	*Endocardial Volume; d - Endocardial Volume; s*
Endocardial %EF	Percent ejection fraction	%	$\frac{Endocardial\ SV}{Endicardial\ Vol;d}\times 100$
Endocardial %FAC	Percent fractional area change	%	$\frac{Endocardial\ area;d - Endocardial\ area;s}{Endicardial\ area;d}\times 100$
LV Mass	LV Mass	mg	$1.05\times(5/6\times Epicardial\ Area;d\times(Epicardial\ Major;d + T;d)) - (5/6\times Endocardial\ Area;d\times (Endocardial\ Major;d))$
Simp Volume; d	Simpson's volume calculation in diastole	μL	$(AreaProx;d + AreaMid;d)\times h + AreaDist;d\times\frac{h}{2}+\frac{\pi}{6}\times h^3$<br>h = Simpson Length in diastole
Simp Volume; s	Simpson's volume calculation in systole	μL	$(AreaProx;s + AreaMid;s)\times h + AreaDist;s\times\frac{h}{2}+\frac{\pi}{6}\times h\text{^}3$<br>h = Simpson Length in diastole
Simp SV	Stroke volume	μL	*Simp Volume; d - Simp Volume; s*
Simp %EF	Ejection fraction	%	*100 * Simp SV / Simp Volume; d*

Supplementary Table S3

GeneNetwork/WebQTL correlation analysis of cardiac gene expression vs LVSD/HF-related traits

	Record ID	Gene Symbol	Description	Mean Expression	LVD		D%EF	
					Correlation	p-Value	Correlation	p-Value
1	ILMN_2856568	**Gpbp1**	Mus musculus GC-rich promoter binding protein 1	9.063	0.897	1.22E-05	0.888	2.29E-05
2	ILMN_1219002	**Usmg5**	Upregulated during skeletal muscle growth 5	8.049	0.924	1.21E-06	0.857	1.20E-04
3	ILMN_2712634	**Nab1**	Mus musculus Ngfi-A binding protein 1	7.026	-0.843	2.20E-04	-0.901	9.26E-06
4	ILMN_2924601	**Klhl17**	Mus musculus kelch-like 17 (Drosophila)	8.672	-0.891	1.92E-05	-0.815	6.20E-04
5	ILMN_1254837	**Rab7**	Mus musculus RAB7, member RAS oncogene family	7.031	-0.857	1.20E-04	-0.844	2.10E-04
6	ILMN_1250201	**Fndc10**	Fibronectin type III domain containing 10 (B930041F14Rik)	7.574	-0.817	5.90E-04	-0.938	2.44E-07
7	ILMN_3163159	**Gng5**	Mus musculus guanine nucleotide binding protein (G protein), gamma 5 subunit	7.813	0.858	1.20E-04	0.815	6.20E-04
8	ILMN_2748205	**Hspb3**	Mus musculus heat shock protein 3	8.589	0.843	2.20E-04	0.813	6.60E-04
9	ILMN_2724570	**Mapk12**	Mus musculus mitogen-activated protein kinase 12	8.106	-0.816	6.10E-04	-0.875	4.96E-05
10	ILMN_2821501	**Zfand6**	Mus musculus zinc finger, AN1-type domain 6	7.536	0.859	1.10E-04	0.803	8.90E-04
11	ILMN_1227012	**Ndufb4**	Mus musculus NADH dehydrogenase (ubiquinone) 1 beta subcomplex 4	8.2	0.837	2.80E-04	0.815	6.10E-04
12	ILMN_1235230	**Pdlim3**	Mus musculus PDZ and LIM domain 3	8.734	-0.842	2.30E-04	-0.806	8.10E-04
13	ILMN_3144289	**Traf3**	Mus musculus Tnf receptor-associated factor 3	8.463	0.832	3.40E-04	-0.821	5.00E-04
14	ILMN_2776899	**Atp5k**	ATP synthase, H+ transporting, mitochondrial F1F0 complex, subunit E	7.113	0.860	1.10E-04	0.795	1.13E-03
15	ILMN_2670230	**Tbrg4**	Mus musculus transforming growth factor beta regulated gene 4	7.428	-0.884	2.87E-05	-0.787	1.43E-03
16	ILMN_2723881	**Hcfc1**	Host cell factor C1	8.766	0.804	8.70E-04	0.832	3.40E-04
17	ILMN_2756008	**Nasp**	Nuclear autoantigenic sperm protein (histone-binding)	7.439	-0.864	8.65E-05	-0.786	1.47E-03
18	ILMN_3133522	**Ppm1d**	Mus musculus protein phosphatase 1D magnesium-dependent, delta isoform	8.126	-0.841	2.40E-04	-0.802	9.40E-04
19	ILMN_2671689	**Cox7b**	Cytochrome c oxidase subunit VIIb	8.63	0.819	5.30E-04	0.810	7.30E-04
20	ILMN_2718416	**Eif3k**	Mus musculus eukaryotic translation initiation factor 3	9.583	0.794	1.17E-03	0.824	4.50E-04
21	ILMN_2680262	**Polr2g**	Mus musculus polymerase (RNA) II (DNA directed) polypeptide G	9.393	0.831	3.50E-04	0.786	1.44E-03
22	ILMN_1242391	**Tmem8**	Mus musculus transmembrane protein 8 (five membrane-spanning domains)	7.027	0.793	1.19E-03	0.805	8.60E-04
23	ILMN_2684279	**Edem2**	ER degradation enhancer, mannosidase alpha-like 2 (9530090G24Rik)	8.636	-0.801	9.50E-04	-0.792	1.23E-03
24	ILMN_2896492	**Jmjd8**	Jumonji domain containing 8 (2610003J06Rik)	8.918	-0.777	1.84E-03	-0.821	5.00E-04
25	ILMN_2868981	**Ehbp1**	Mus musculus EH domain binding protein 1	8.526	0.827	4.10E-04	0.764	2.57E-03
26	ILMN_2972645	**Pdzd11**	Mus musculus PDZ domain containing 11	8.285	-0.765	2.49E-03	-0.854	1.40E-04
27	ILMN_2655429	**C530015C18**	Hypothetical protein C530015C18	7.95	-0.838	2.70E-04	-0.748	3.68E-03
28	ILMN_2966826	**Ttll1**	Mus musculus tubulin tyrosine ligase-like 1	9.286	0.787	1.41E-03	0.783	1.56E-03
29	ILMN_1257503	**Tm9sf2**	Mus musculus transmembrane 9 superfamily member 2	7.024	0.786	1.45E-03	0.777	1.87E-03
30	ILMN_2698606	**Tmed4**	Transmembrane p24 trafficking protein 4 (1110014L17Rik)	8.147	-0.779	1.78E-03	-0.786	1.46E-03
31	ILMN_1221702	**Plaa**	phospholipase A2, activating protein	9.027	0.803	9.00E-04	0.755	3.14E-03
32	ILMN_2692615	**Tgm2**	Transglutaminase 2, C polypeptide	11.845	-0.763	2.59E-03	-0.802	9.30E-04
33	ILMN_2687032	**Fbxo31**	Mus musculus F-box protein 31	8.462	-0.784	1.54E-03	-0.761	2.72E-03
34	ILMN_2591917	**Akr1b3**	Aldo-keto reductase family 1, member B3 (aldose reductase)	9.977	0.828	3.90E-04	0.7179	6.73E-03
35	ILMN_1215862	**Cxcl9**	Chemokine (C-X-C motif) ligand 9	7.752	0.771	2.16E-03	0.771	2.14E-03
36	ILMN_1223614	**Rbpms**	RNA binding protein gene with multiple splicing	10.15	-0.769	2.27E-03	-0.773	2.05E-03
37	ILMN_2771219	**Pdlim5**	PDZ and LIM domain 5 (1110001A05Rik)	11.826	-0.785	1.51E-03	-0.757	3.00E-03
38	ILMN_2666406	**Prrc1**	proline-rich coiled-coil 1 (1190002C06Rik)	9.173	-0.788	1.37E-03	-0.748	3.69E-03
39	ILMN_2605004	**Ndufb9**	Mus musculus NADH dehydrogenase (ubiquinone) 1 beta subcomplex, 9	11.241	0.768	2.34E-03	0.768	2.33E-03
40	ILMN_3126933	**Tacc1**	Mus musculus transforming, acidic coiled-coil containing protein 1	8.472	-0.754	3.23E-03	-0.796	1.12E-03
41	ILMN_1249116	**Cpsf3l**	Mus musculus cleavage and polyadenylation specific factor 3-like	7.78	-0.762	2.71E-03	-0.760	2.81E-03
42	ILMN_2702197	**Pcbp1**	Poly(rC) binding protein 1	10.336	-0.781	1.69E-03	-0.731	5.19E-03
43	ILMN_1230271	**Anp32a**	Mus musculus acidic (leucine-rich) nuclear phosphoprotein 32 family, member A	7.812	0.774	1.99E-03	0.744	4.00E-03
44	ILMN_1252302	**Taf6**	TAF6 RNA polymerase II, TATA box binding protein (TBP)-associated factor	7.257	0.759	2.87E-03	0.762	2.65E-03
45	ILMN_2510364	**Trip12**	Thyroid hormone receptor interactor 12	7.455	0.805	8.50E-04	0.705	8.50E-03
46	ILMN_2928573	**Rev1**	Mus musculus REV1 homolog (S. cerevisiae)	7.205	-0.747	3.75E-03	-0.795	1.13E-03
47	ILMN_2973097	**Coq3**	Mus musculus coenzyme Q3 homolog, methyltransferase (yeast)	7.495	0.774	2.02E-03	0.742	4.17E-03
48	ILMN_2859978	**Papss1**	Mus musculus 3'-phosphoadenosine 5'-phosphosulfate synthase 1	8.581	-0.761	2.73E-03	-0.758	2.91E-03
49	ILMN_2883952	**YdjC**	YdjC homolog (bacterial) (1810015A11Rik)	7.148	-0.756	3.08E-03	-0.761	2.71E-03
50	ILMN_3008361	**Bmper**	Mus musculus BMP-binding endothelial regulator	7.414	-0.744	4.01E-03	-0.785	1.49E-03

The 50 top correlates common for the LVD and ΔEF traits (correlation coefficient ≥0.74) for normalized gene expression level >7.

P-value provided according to Pearson's product-moment correlations.

Table S4 List of functional SNPs corresponding to the QTL located on Chromosome 12 associated with the myocardial rupture trait

Gene	dbSNP	Genomic Location	Ref	129S1/ SvImJ	A/J	C57BL/ 6NJ	CAST/EiJ	NOD/ ShiLtJ	NZO/ HlLtJ	PWK/PhJ	WSB/EiJ	Consequence Type	Amino acids	Protein position	Impact	SIFT
**Nrxn3**	rs231902337	12:90283564	*G*	-	-	-	-	-	-	A	-	Splice region variant			low	
**Cep128**	rs227456595	12:91159065	*C*	-	-	-	G	-	-	G	-	Splice donor variant			high	
**Cep128**	rs249098074	12:91183657	*G*	-	-	-	-	-	-	T	-	Splice region variant			low	
**Cep128**	rs261503899	12:91213702	*C*	-	-	-	-	-	-	T	-	Missense variant	S/N	889	moderate	tolerated (0.57)
**Cep128**	rs48798187	12:91213755	*A*	-	-	-	-	-	-	G	-	Splice region variant			low	
**Cep128**	rs48026157	12:91234161	*A*	-	-	-	C	-	-	C	-	Missense variant	H/Q	811	moderate	tolerated (1)
**Cep128**	rs46662645	12:91255722	*A*	-	-	-	-	-	-	T	-	Missense variant	D/E	742	moderate	tolerated (0.43)
**Cep128**	rs261762568	12:91260124	*G*	-	-	-	T	-	-	-	-	Missense variant	D/E	690	moderate	tolerated (0.5)
**Cep128**	rs51557552	12:91260575	*T*	-	-	-	C	-	-	C	-	Missense variant	N/S	540	moderate	tolerated (0.52)
**Cep128**	rs46446778	12:91266888	*T*	-	-	-	C	-	-	C	-	Missense variant	T/A	469	moderate	tolerated (1)
**Cep128**	rs262007891	12:91294082	*G*	-	-	-	-	-	-	C	-	Missense variant	P/A	336	moderate	tolerated (0.08)
**Cep128**	rs46978148	12:91294173	*T*	-	-	-	C	-	-	C	-	Splice region variant			low	
**Cep128**	rs252980304	12:91299035	*C*	-	-	-	T	-	-	T	-	Missense variant	V/I	268	moderate	tolerated (0.32)
**Cep128**	rs217950132	12:91320001	*T*	-	-	-	G	-	-	G	-	Splice region variant			low	
**Cep128**	rs266165675	12:91366444	*C*	-	-	-	-	-	-	T	-	Missense variant	G/R	16	moderate	tolerated (0.92)
**Cep128**	rs49587405	12:91370201	*G*	-	-	-	A	-	-	A	-	Splice region variant			low	

Table S5 List of functional SNPs corresponding to the QTL located on Chromosome 5 associated with the LVD trait

Gene	dbSNP	Genomic Location	Ref	129S1/SvImJ	A/J	C57BL/6NJ	CAST/EiJ	NOD/ShiLtJ	NZO/HlLtJ	PWK/PhJ	WSB/EiJ	Consequence Type	Amino acids	Protein position	Impact	SIFT
**Tfip11**	rs32109670	5:112333231	*G*	-	-	-	A	-	-	-		Missense variant	E/K	358	moderate	tolerated (0.39)
**Tfip11**	rs33218387	5:112333336	*G*	-	-	-	-	-	A	-	A	Missense variant	A/T	393	moderate	tolerated (0.63)
**Srrd**	rs234879464	5:112339730	*T*	-	-	-	G	-	-	-		Missense variant	S/R	169	moderate	tolerated (0.21)
**Gm6583**	rs32113318	5:112354764	*C*	-	-	-	T	-	-	-		Missense variant	C/T	358	moderate	tolerated (0.51)
**Gm6583**	rs264081076	5:112355358	*T*	-	-	-	C	-	-	C	-	Missense variant	K/R	160	moderate	tolerated (0.8)
**Gm6583**	rs32113322	5:112355547	*G*	-	-	-	A	-	-	-		Missense variant	A/V	97	moderate	tolerated (0.88)
**Gm6583**	rs258837611	5:112355749	*C*	-	-	-	T	-	-	-		Missense variant	G/R	30	moderate	tolerated (0.97)
**Hps4**	rs253490750	5:112349378	*C*	-	-	-	-	-	-	-	T	Missense variant	R/C	76	moderate	tolerated(0.05)
**Hps4**	rs239835610	5:112368976	*C*	-	-	-	T	-	-	-		Splice region variant	-	-	low	
**Hps4**	rs32107636	5:112370143	*G*	-		-	A	-	-	-		Missense variant	C/Y	334	moderate	tolerated (0.16)
**Hps4**	rs228327059	5:112374979	*C*	-	-	-	-	-	-	-	T	Missense variant	L/F	568	moderate	deleterious (0)
**Hps4**	rs32112489	5:112377955	*A*	-	-	-	T	-	-	-		Splice region variant	-	-	low	
**Sez6l**	rs32114817	5:112422009	*T*	-	-	-	C	-	-	-		Stop retained variant	-	-	low	
**Sez6l**	rs32116976	5:112425616	*C*	-	-	-	-	-	-	-	T	Splice region variant	-	-	low	
**Sez6l**	rs32122208	5:112472926	*G*	-	-	-	-	-	-	-	A	Splice region variant	-	-	low	
**Sez6l**	rs32118052	5:112475023	*G*	C	-	-	-	C	-	C	C	Splice region variant	-	-	low	
**Sez6l**	rs32118955	5:112475412	*C*	T	-	-	-	T	-	-	T	Missense variant	C/N	91	moderate	tolerated (0.44)
**Myo18b**	rs212801037	5:112775403	*A*	-	-	-	T	-	-	-	-	Splice region variant	-	-	low	
**Myo18b**	rs47968507	5:112802426	*G*	C	-	-	-	C	C	-	-	Missense variant	L/V	1535	moderate	tolerated (0.18)
**Myo18b**	rs46076017	5:112809682	*T*	C	C	-	-	C	C	C	-	Missense variant	T/A	1472	moderate	tolerated (0.87)
**Myo18b**	rs237692588	5:112837808	*T*	-	-	-	C	-	-	-	-	Missense variant	T/A	1092	moderate	tolerated (0.6)
**Myo18b**	rs48666049	5:112837826	*G*	-	-	-	C	-	-	C	-	Missense variant	Q/E	1086	moderate	tolerated (0.8)
**Myo18b**	rs234142160	5:112858503	*C*	-	-	-	T	-	-	-	-	Missense variant	R/Q	847	moderate	tolerated (1)
**Myo18b**	rs48271099	5:112871438	*C*	-	-	-	T	-	-	-	-	Missense variant	V/M	658	moderate	tolerated (0.06)
**Myo18b**	rs260627251	5:112871446	*G*	-	-	-	T	-	-	-	-	Missense variant	T/K	655	moderate	tolerated (0.12)
**Myo18b**	rs220128630	5:112871546	*C*	-	-	-	T	-	-	-	-	Missense variant	V/I	622	moderate	tolerated (0.09)
**Myo18b**	rs238856395	5:112871552	*C*	-	-	-	T	-	-	T	-	Missense variant	V/I	620	moderate	tolerated (0.69)
**Myo18b**	rs51366779	5:112873984	*A*	-	-	-	C	-	-	C	-	Missense variant	V/G	514	moderate	tolerated-LC (0.44)
**Myo18b**	rs45640773	5:112874114	*C*	-	-	-	T	-	-	-	-	Missense variant	A/T	471	moderate	tolerated-LC (0.18)
**Myo18b**	rs244439177	5:112874302	*G*	-	-	-	A	-	-	-	-	Missense variant	P/L	408	moderate	tolerated-LC (0.08)
**Myo18b**	rs582288427	5:112874336	*G*	-	-	-	A	-	-	-	-	Missense variant	P/S	397	moderate	tolerated-LC (0.41)
**Myo18b**	rs584773560	5:112874338	*C*	-	-	-	T	-	-	-	-	Missense variant	G/E	396	moderate	tolerated-LC (0.37)
**Myo18b**	rs585945270	5:112874344	*G*	-	-	-	A	-	-	-	-	Missense variant	P/L	394	moderate	tolerated-LC (0.19)
**Myo18b**	rs580478289	5:112874362	*G*	-	-	-	A	-	-	-	-	Missense variant	A/V	388	moderate	tolerated-LC (0.22)
**Myo18b**	rs249974165	5:112874363	*C*	-	-	-	A	-	-	A	-	Missense variant	A/S	388	moderate	tolerated-LC (0.43)
**Myo18b**	rs212290244	5:112874384	*A*	-	-	-	C	-	-	-	-	Missense variant	S/P	1369	moderate	tolerated-LC (0.73)
**Myo18b**	rs47749211	5:112874531	*G*	-	-	-	C	-	-	-	-	Missense variant	R/G	332	moderate	tolerated-LC (0.01)
**Myo18b**	rs51932075	5:112874585	*A*	-	-	-	G	-	-	G	-	Missense variant	W/R	314	moderate	tolerated-LC (1)
**Myo18b**	rs581501658	5:112874614	*G*	-	-	-	A	-	-	-	-	Missense variant	P/L	304	moderate	tolerated-LC (0.23)
**Myo18b**	rs257889290	5:112874803	*A*	-	-	-	T	-	-	T	-	Missense variant	I/N	241	moderate	tolerated-LC (0.09)
**Myo18b**	rs33706581	5:112875024	*A*	-	-	-	C	-	-	C	-	Missense variant	D/E	167	moderate	tolerated-LC (1)

Table S6

The Jackson Laboratory nomenclature of the CC founder and RI strains used in this study

ARC/GENIAD Strain Code	JAX Strain Code
129X1/SvJArc	129S1/SvImJ
A/JArc	A/J
C57BL/6JArc	C57BL/6J
NOD/ShiLtJArc	NOD/ShiLtJ
BEM_AG	CC032
BOM_GB	CC042
BOON_HF	CC008
CIS_AD	CC024
DAVIS_BA	CC012
DOD_AH	N/A
DONNELL_HA	N/A
FEW_FD	CC025
FIM_DF	CC061
FIV_AC	N/A
GALASUPREME_CE	N/A
GIG_EF	CC013
GIT_GC	N/A
HAZ_FE	N/A
JUNIOR_GB	CC020
LUZ_FH	N/A
MERCURI_HF	N/A
NUK_AC	CC010
PAT_CD	N/A
PEF_EC	N/A
POH_DC	N/A
PUB_CD	CC056
SAT_GA	CC016
TAS_FE	CC026
TOFU_FB	CC027
TOP_DA	CC023
WAB2_DH	CC031
YOX_DE	N/A

N/A – strain not maintained at the Jackson Laboratory depository

Table S7

Abbreviations of the mouse strains employed in this publication

ARC strains	Abbreviation
129X1/SvJArc	129X1
A/JArc	AJ
C57BL/6JArc	B6
NOD/ShiLtJArc	NOD
**CC RI strains**	
BEM_AG	BEM
BOM_GB	BOM
BOON_HF	BOON
CIS_AD	CIS
DAVIS_BA	DAVIS
DOD_AH	DOD
DONNELL_HA	DONNELL or DON
FEW_FD	FEW
FIM_DF	FIM
FIV_AC	FIV
GALASUPREME_CE	GALASUPREME or GALA
GIG_EF	GIG
GIT_GC	GIT
HAZ_FE	HAZ
JUNIOR_GB	JUNIOR
LUZ_FH	LUZ
MERCURI_HF	MERCURY
NUK_AC	NUK
PAT_CD	PAT
PEF_EC	PEF
POH_DC	POH
PUB_CD	PUB
SAT_GA	SAT
TAS_FE	TAS
TOFU_FB	TOFU
TOP_DA	TOP
WAB2_DH	WAB2
YOX_DE	YOX

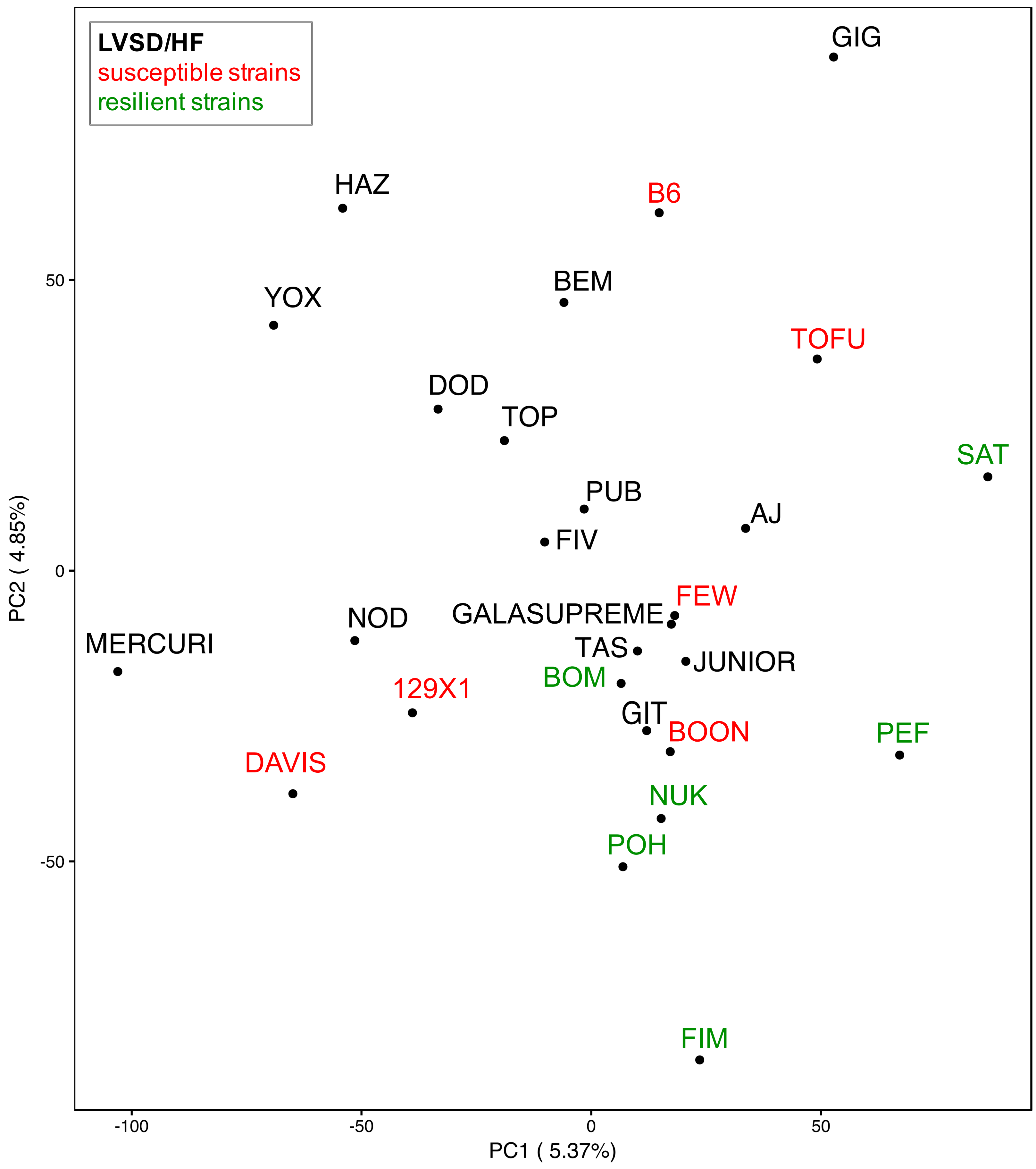

Supplementary Figure S1. Principal Component Analysis (PCA) on whole-transcriptome profiles of 27 strains employed in this study. The plot depicts a principal component analysis of the transcriptomes of uninjured hearts across 27 selected RI CC strains. The first principal component (PC1) explains 5.37% of the observed variation across strains while the second principal component (PC2) explains 4.85% of the variation. LVSD/HF resilient strains are highlighted in green, while LVSD/HF susceptible strains are highlighted in red.

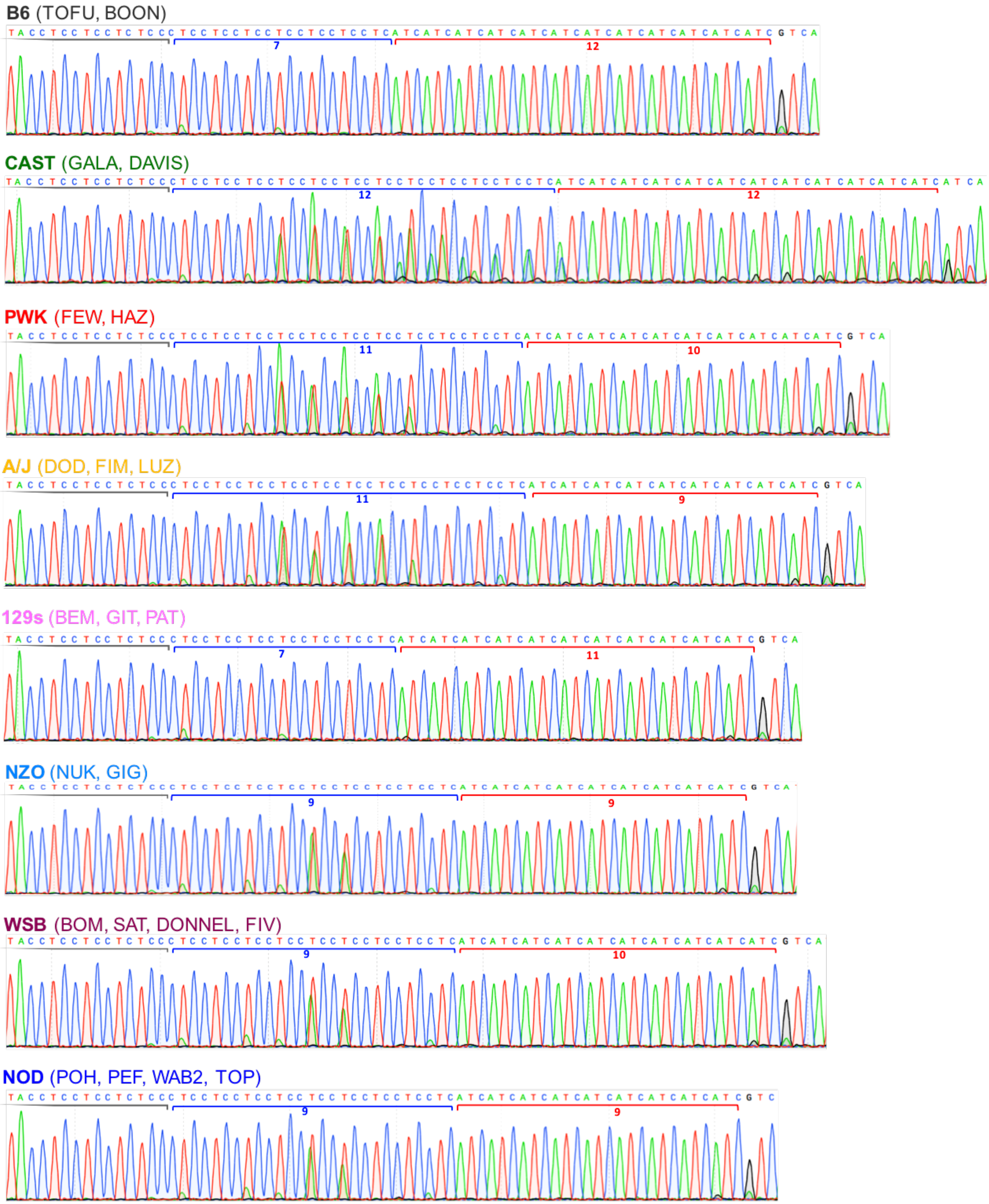

Supplementary Figure S2. Intronic polymorphism in the *Myo18b* region associated with the LVD trait.

Representative chromatograms of the 8 haplotypes in the intronic region (Chr5: 112856370-112856440) capturing microsatellite variation corresponding to polymorphisms rs578974233, rs582659088, rs585674637 and rs226166519.

Forward primer: 5'-AAGCCCATGAATGCACATTGG; reverse primer: 5'-AGACTTGGTGGAAGAGGCAA

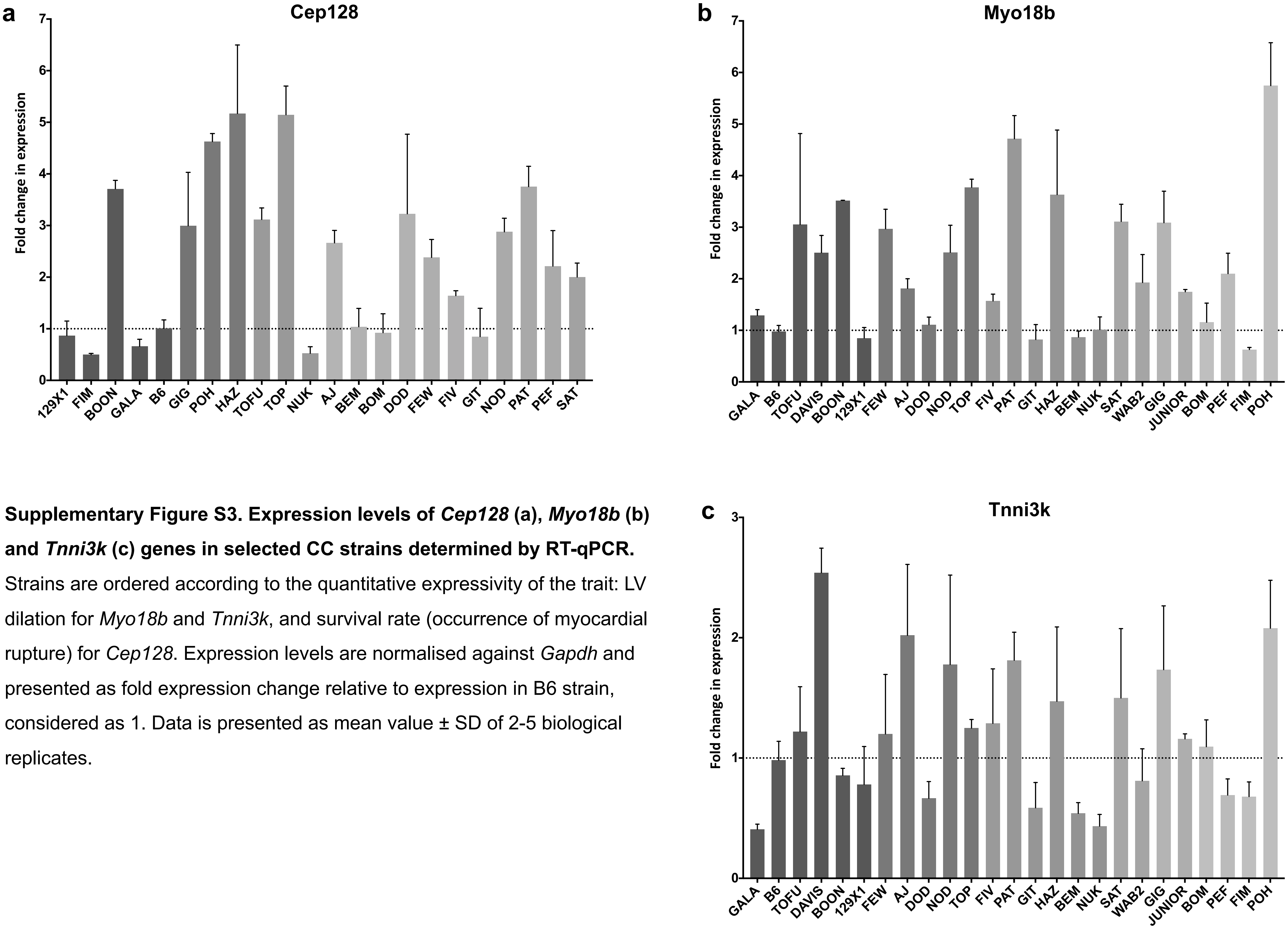

Supplementary Figure S3. Expression levels of *Cep128* (a), *Myo18b* (b) and *Tnni3k* (c) genes in selected CC strains determined by RT-qPCR. Strains are ordered according to the quantitative expressivity of the trait: LV dilation for *Myo18b* and *Tnni3k*, and survival rate (occurrence of myocardial rupture) for *Cep128*. Expression levels are normalised against *Gapdh* and presented as fold expression change relative to expression in B6 strain, considered as 1. Data is presented as mean value ± SD of 2-5 biological replicates.
